# Graph Neural Networks for Polymer Characterization and Property Prediction: Opportunities and Challenges

**DOI:** 10.1021/acs.jcim.5c02421

**Published:** 2026-01-29

**Authors:** Hector Medina, Rachel Drake

**Affiliations:** School of Engineering, 5199 Liberty University, Lynchburg, Virginia 24515, United States

**Keywords:** graph neural networks, polymer characterization, property prediction, density functional theory, molecular dynamics, coarse-graining, datasets, machine learning

## Abstract

Using machine learning to accelerate the characterization and prediction of properties of many-molecule systems, such as polymers, is appealing, yet challenging. Polymers are large, complex molecules that have unique properties and potential applications in a wide range of industries. Their potential in advancing fields such as ion-transport polymer for energy storage, lightweighting of structural materials, bioinspired multifunctional materials, etc., provide enough impetus for accelerating the discovery of novel polymeric materials. However, mathematical mapping and the consequent manipulation of polymer structures are still challenging tasks due to their complex configuration and the smorgasbord of motifs encountered naturally and in engineering materials. Traditional methods of polymer structure mapping and property prediction at multiscale domains can include approaches such as Density Functional Theory, Molecular Dynamics, and Finite Element Analysis, which can be time-consuming and computationally expensive. The promise of machine learning to accelerate these tasks is appealing, and currently, researchers are pursuing the development of architectures and composition approaches to accomplish this. Here we discuss the current state of the knowledge on the use of Graph Neural Networks, and related architectures, being developed and/or used for the characterization and prediction of properties of polymers. Many challenges still exist such as the lack of sufficient and comprehensive data sets. To address these issues, efforts are being pursued—such as the so-called *CRIPT* (Community Resource for Innovation in Polymer Technology) led by a lab consortium that includes representations from private industry, academia, government, and others. We conclude that even though this field is young it has both momentum and promise. The current challenges that must be overcome are also addressed.

## Graphical abstract

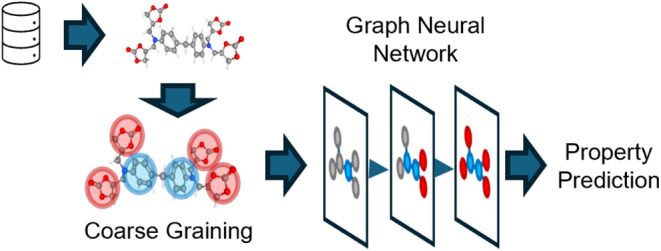

## 1 Introduction

Polymers—topologically complex compositions of chains of repeating molecular units—play an important role in materials, electronics, and biomedical devices due to their unique properties and versatility. Understanding and predicting their broad chemical structures is vital for the creation of applications in energy storage, lightweight structural materials, and bioinspired multifunctional materials.^1–13^ However, the complexity of polymers with repeat units, diverse topologies, and multiscale interactions presents challenges for property prediction and material design.^14–19^ Traditional methods such as Density Functional Theory (DFT), Molecular Dynamics (MD), and Finite Element Analysis (FEA) have been used for polymer characterization.^20–27^ These are computational approaches ranging from first-principle methods like DFT to atomistic simulations such as MD and continuum scale techniques like FEA to characterize polymer behavior. DFT offers information on electronic structures;^28–30^ however, it is computationally demanding for large polymer systems.^31–35^ MD simulations give thorough dynamics behavior, but are limited by system size and simulation times.^18,36–38^ FEA excels at modeling the mechanical behavior of materials, but struggles with the geometrical intricacy of polymers.^39–41^ Although accurate, these methods face scalability issues due to computational requirements to model large polymer systems.^42–44^

Machine learning (ML) is a promising alternative for polymer characterization using data-driven insights to predict properties.^45–47^ Random forests (RF),^48–50^ support vector machines (SVM),^51–54^ and artificial neural networks (ANN)^55–58^ are some techniques that have shown promise for the identification of patterns and relationships in polymer data.^59–63^ However, these methods often require tedious feature engineering and may not fully encode the relational or hierarchical nature of polymer structures.^64–67^

Graph Neural Networks (GNNs) are a step forward in polymer science using ML. Rather than replacing physics based methods such as DFT, MD, or FEA, GNNs function as data driven surrogate models trained on their outputs, extending these simulations to larger chemical spaces. GNNs can naturally handle the graph-like structures of polymers by representing molecular structures as graphs with atoms as nodes and bonds as edges.^68–71^ GNNs extend traditional neural networks (NNs) and convolutional neural networks (CNNs) to operate on graph-structured data that can learn the local neighborhood information on each node through message-passing mechanisms.^72–77^ Graph-based inputs allow these models to capture structural patterns across multiple scales, from monomer chemistry to polymer-chain architecture, providing a unified way to learn relationships embedded within the topology. In contrast to traditional ML approaches, GNNs are invariant to atom ordering and can handle the complex connectivity of polymer chains.^69,78,79^ GNNs have shown promise in many areas, including social network analysis, bioinformatics, and materials science, since they can capture complex structural information in graphs.^80–89^ GNN architectures such as Graph Convolutional Networks (GCNs), Message Passing Neural Networks (MPNNs), and Graph Attention Networks (GATs) have been modified to improve polymer property prediction.^90–97^ (We provide a somewhat comprehensive overview of the GNN architectures in Section 2.)

The tasks of modeling polymer structures and predicting their properties using GNN are not without challenges. One of them is the lack of suitable data sets that match the smorgasbord variety of polymer’s chemistry, structure, and properties.^98–100^ Furthermore, even if data mining is carried out to consolidate information from various sources, other issues arise, such variation of levels of accuracy, distinct experimental setups, various synthesis methods, etc., from different data sets.^101^ Projects such as the Community Resource for Innovation in Polymer Technology (CRIPT) are attempting to overcome some of those challenges. (In Section 3, we provide a summary of some existing data sets along with some remaining challenges.) A further unresolved challenge in polymer informatics is that most current GNN models operate solely on chemical structure and largely neglect morphology, processing history, and environmental conditions, factors that critically shape real polymer behavior. Highlighting this gap helps motivate the need for future GNN architectures capable of encoding multiscale and processing-dependent information.

Keys to overcoming challenges related to the proper multiscale representation of polymers is the proper composition—and corresponding embedding—of groups of atoms into larger “pseudo components”, a concept referred to as “coarse graining” (CG).^102–105^ More recent advances in polymer-specific GNN architectures have incorporated coarse-grained graph representations to connect atomistic level information with macroscopic polymer properties.^106–108^ Several challenges still remain with CG, especially related to the trade-off of accuracy versus computational cost. A nonexhaustive list of CG models with their corresponding key features are summarized in Section 4.

Applications of GNNs in polymer research span a wide range of property prediction tasks. These include fundamental thermophysical properties such as glass transition temperature, melting point, solubility, and density, as well as performance-relevant attributes like dielectric constant, mechanical strength, permeability, and viscosity.^109–111^ Beyond bulk properties, GNNs have been applied in specialized domains such as gas separation membranes, polymer electrolytes for batteries, and functional polymers for drug delivery or organic electronics.^106^ Importantly, GNN-based models have demonstrated the ability to generalize across diverse polymer classes, learning transferable chemical representations that outperform conventional machine learning approaches on large databases. Some advances in applying GNNs to polymer property prediction are also reviewed here, both their strengths and limitations. Specific polymer property prediction tasks and associated challenges are discussed in Section 5.

Finally, we provide, under the Discussion and Overlook section, an overview of some interpretability methods used and highlight the key innovations that define emerging trends and future opportunities in the general area of polymer informatics. While several existing reviews discuss ML for polymers or GNNs for molecules and materials more broadly, fewer works explicitly examine how polymer-specific graph representations, data limitations, and multiscale considerations shape the use of GNNs in polymer informatics. This review synthesizes polymer-focused GNN works by overviewing representation choices (repeat-unit/periodic and coarse-grained graphs), data set and infrastructure constraints, and application-driven modeling across key polymer property domains, while highlighting open challenges in data quality/coverage and in capturing morphology and processing effects. To our knowledge, no other review has focused entirely on GNN-based polymer informatics, as outlined.

## 2 GNN Architectures

In this section, we review some of the common GNN architectures used to model complex polymer systems. The models are grouped according to their core architectural classes, MPNNs, GCNs, and GATs. Each class reflects different strategies for encoding graph input, message propagation, and handling polymer-specific topologies. Table 1 provides an overview of representative GNN types and models, including their training data, polymer families, and target properties. Compared with traditional neural networks that operate on fixed-size vectors, GNNs naturally process variable graph structures and therefore support edge-, node-, and graph-level prediction tasks.^68,69,87,112^ Unlike traditional NNs that process fixed-size vectors, GNNs can handle data with diverse structures, making them suitable for edge, node, and graph prediction.^72,113–115^ Across these architectures, message passing layers update node and edge features based on local neighborhoods, while readout layers aggregate node representations into a graph-level embedding suitable for property prediction.^87,116,117^ Figure 1 provides a schematic overview of the basic GNN architectures, illustrating their shared message-passing and readout components. GNNs can capture features such as repeat units, ring structures, and periodic graph patterns supporting improved generalization in the prediction of polymer properties.

**Figure 1:**
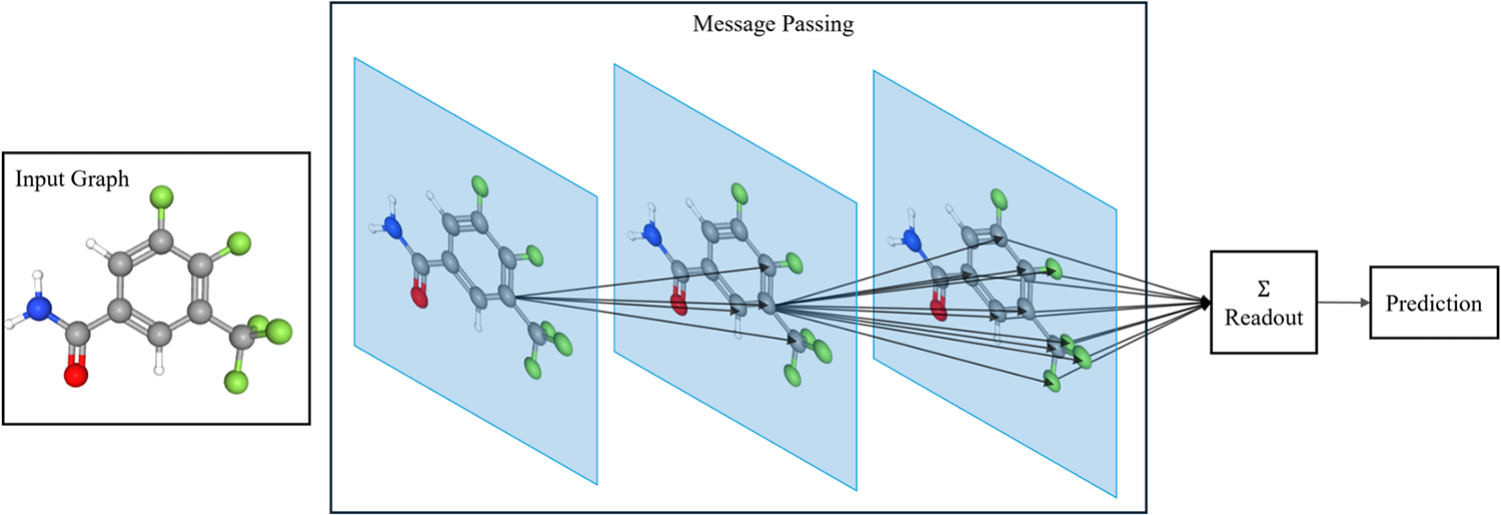
General architecture of a GNN for molecular property prediction. The input molecular graph is processed through multiple message passing layers, where the node embeddings are iteratively updated by propagating information from neighboring atoms. These updated representations are then combined by a readout function (e.g., average or sum) to produce a graph-level embedding, which is fed into a downstream prediction layer.

**Table 1:** GNN Architectures

model	type	training data	polymer family	target properties
PU-gn-exp^106^	MPNN	697 polymers, collected from literature	conjugated semiconducting polymers	mobility, HOMO, LUMO
wD-MPNN^121^	MPNN	42,966 copolymers, computational (xTB/DFT)	conjugated copolymers	IP, EA
GH-GNN^124^	MPNN	2500–2800 polymer–solvent pairs, experimental	homopolymer–solvent mixtures	activity coefficient at infinite dilution
polyGNN^126^	MPNN	13,388 polymers, computational and experimental	diverse synthetic polymers	multiproperty: electronic, optical, thermal, mechanical, solubility
St. John MPNN^127^	MPNN	91,000 molecules and oligomers, computational (DFT)	OPV candidates	HOMO, LUMO, excitation energy
Periodic D-MPNN^123^	MPNN	15,219 data points, experimental and DFT	homopolymers	atomization energy, bandgap, EA, dielectric, *T*_g_
GNN-A/B	MPNN	372 experimental polyimides (8,205,096 virtual candidates screened)	polyimides	*T*_g_
sGNN^108^	MPNN	20,000 MP2 conformations, computational	PEG, PE fragments	bonding potential energy
Multitask GNN^147^	GCN	876 polymers (5 ns MD), 117 polymers (50 ns MD)	polymer electrolytes	ionic conductivity, diffusivity
Chem-DAGNN^148^	GCN	687 polyimides, experimental	polyimides	*T*_g_
Park GCN^149^	GCN	2687 Organic Polyamides	polyamides	*T*_g_, *T*_m_, density, elastic modulus
Hu GCN^133^	GCN	300–600 polymers, experimental	homopolymers	*T*_g_
Hickey GCN^134^	GCN	7558 polymers, experimental	general polymers	*T*_g_
Zeng GCNN^110^	GCN	1073 polymers, computational (DFT)	organic polymers	dielectric constant, bandgap
SweetNet^150^	GCN	19,775 glycans, experimental	glycans	taxonomy, immunogenicity, pathogenicity, viral glycan bonding
Kimmig GCNN^135^	GCN	2813 nanoparticle measurements, experimental	poly(methacrylates)	nanoparticle size
Volgin GCNN^151^	GCN	6,726,950 synthetic (QSPR) and 214 experimental polyimides	polyimides	*T*_g_
GATBoost^144^	GAT	235 polymers, experimental	acid containing polymers	*T*_g_
POLYMERGNN^111^	GAT	296 polymers, experimental	polyesters	*T*_g_, intrinsic viscosity
GNNs^107^	MPNN, GAT, GCN	1313 glycans, 15,778 peptides, experimental	glycans, peptides	immunogenicity, taxonomy, minimum inhibitory concentration

This section outlines the core computational structure and polymer-specific adaptations of GNN models, with performance results deferred to a later section. For each class, we organize the contributions by their input representation, GNN design choices, and output strategies.

### 2.1 Message Passing Neural Networks

Message Passing Neural Networks (MPNNs) operate on molecular graphs by iteratively exchanging information between nodes based on both node and edge features.^91,118–120^ Given a graph *G* = (*V*, *E*), where *V* is the set of nodes and *E* is the set of edges, MPNNs proceed in two phases: a message-passing phase and a readout phase. During the message passing phase each node *v* has a feature vector *h*_*v*_^*t*^ in iteration *t*. For each neighbor *w* ∈ *N*(*v*), the edge message from *w* to *v* is computed by

1 $$m_{v,w}^{t}=M^{t}\left(h_{w}^{t},e_{v,w}\right)$$

then all incoming messages are aggregated at *v* via

2 $${\mathrm{m̃}}_{v}^{t}=\sum_{w\in N\left(v\right)}m_{v,w}^{t}$$

and *v*’s hidden state is updated

3 $$h_{v}^{t+1}=U^{t}\left(h_{v}^{t},{\mathrm{m̃}}_{v}^{t}\right)$$

A graph-level feature vector is obtained by applying a readout (e.g., summation or averaging) over *h*_*vv*∈*V*_^*T*^, for *T* iterations. All learnable parameters in functions *M* ^*t*^, update functions *U* ^*t*^, and the final readout stage are trained from the beginning to the end to minimize loss in a set of labeled graphs. MPNNs serve as a flexible backbone in polymer prediction tasks due to their customizable message and update functions, which are useful in capturing polymer-specific periodicity, stoichiometry, and topological motifs.

The following models demonstrate how the MPNN framework has been extended to predict a wide range of polymer properties including glass transition temperature (*T*_g_), ionic conductivity, diffusivity, electronic structure descriptors, activity coefficients, and force field energy terms. Although detailed benchmarking and interpretability results are deferred to later sections, this section emphasizes architecture design and graph inputs.

MPNNs are adapted in many ways to handle the structural complexity inherent to polymer systems. Several approaches incorporate directed and weighted propagation, where weights encode the frequency or importance of connections within input graphs.^121–123^ MPNNs have also been extended to multigraph systems, allowing interactions between multiple molecular components.^124,125^ In these architectures, each graph is initialized with its own node, edge, and global attributes. Separate message-passing layers are first applied to each graph, and their intermediate embeddings are pooled and passed to a mixture-level GNN, which demonstrates how hierarchical message passing scales naturally to multicomponent systems. Periodicity, localized subgraphs, and repeat units are also addressed by constructing periodic graph inputs and adding virtual edges between terminal nodes, enabling the MPNN to preserve local environments, as demonstrated in Figure 2.^108,126^ Multitask is further implemented by concatenating the pooled embeddings with a task selector vector, one-hot identifiers that condition a shared multilayer perceptron (MLP) to produce multiple outputs from a single model. CG graph representations use higher-level structural subunits rather than individual nodes using preprocessing to extract and store these units.^106^ Each subunit is encoded as a lower-dimensional node embedding, and the resulting graph is passed through an MPNN based on the gn-exp framework.^125^ Additional architectural refinements focus on node update mechanisms such as replacing simple feed-forward updated with GRU-based recurrent updates, that enables iterative refinement of node states during message passing in which information accumulates across multiple message-passing steps.^127–129^ Finally, physics guided adaptations have been developed. One approach constructs localized bond-centered subgraphs defined over internal coordinates, enabling the model to decouple bonding energies from nonbonded interactions and scale linearly with system size.^108^

**Figure 2:**
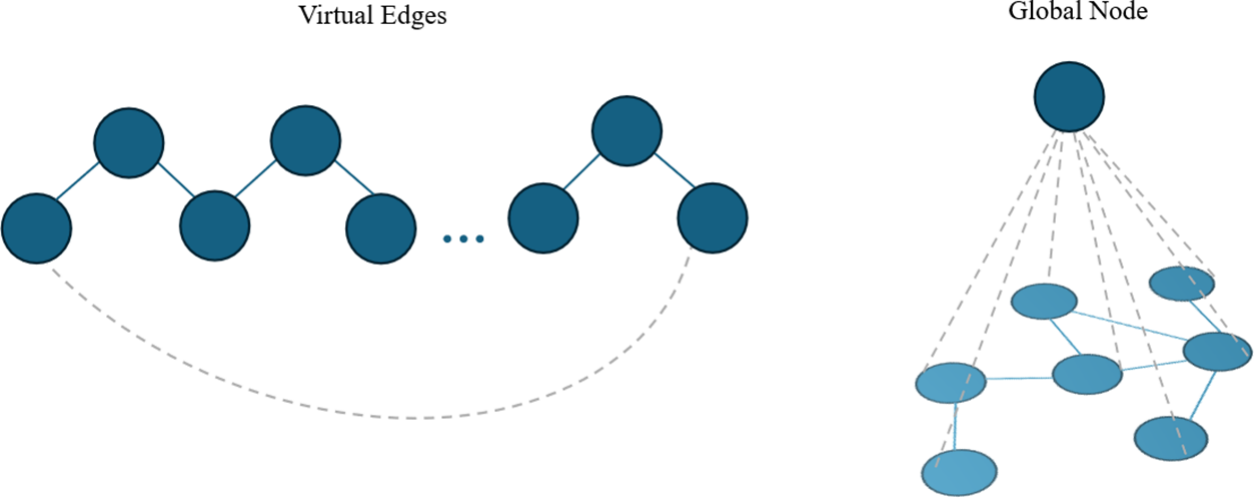
Two graph augmentations in polymer GNN architectures: a virtual edges connecting distant nodes to encode periodic or long-range interactions (left), and a global node connected to all nodes to capture system-level or graph-wide information (right).

MPNN-based architectures have been adapted to retrieve polymer-specific features such as periodicity, stoichiometry, and chain-level structure. These adaptations include the use of directed and weighted edges, repeat-unit graphs, virtual periodic bonds, PU inputs, and message-passing schemes optimized for high-throughput 2D inputs. Some models also introduce input augmentation for translation invariance of repeat units and support multitask learning via property-specific conditioning. Though the basic mechanism of message-passing remains unchanged, these enhancements demonstrate the adaptability of MPNNs to model local chemical properties as well as global polymer topology.

### 2.2 Graph Convolutional Networks (GCN)

Graph Convolutional Networks (GCNs) are NNs that operate on graph-structured inputs.^90,130–132^ Given a graph *G* = (*V*, *E*), a GCN learns the representations of nodes by aggregating information from the node’s neighbors. Let *Ã* = *A* + *I* be the adjacency matrix of the graph *G* with added self-loops, and let D̃ be the corresponding degree matrix. Let *X* be the initial input node feature matrix. The first layer of a GCN is represented as

4 $$H^{\left(1\right)}=\sigma \left({\mathrm{D̃}}^{-1/2}Ã{\mathrm{D̃}}^{-1/2}\mathrm{XW}\right)$$

Here, *W* is a learnable weight matrix, and σ is a nonlinear activation function. The output of the *k*-th layer output of the GCN is given by

5 $$H^{\left(k\right)}=\sigma \left({\mathrm{D̃}}^{-1/2}Ã{\mathrm{D̃}}^{-1/2}H^{\left(k-1\right)}W^{\left(k\right)}\right)$$

For *K* layers, node embeddings are aggregated using a readout function to produce a graph-level feature vector. All parameters are trained from start to finish, minimizing task-specific loss on a set of labeled graphs.

GCNs are used in polymer informatics due to their efficiency and flexibility. This section groups GCN-based models by representational and architectural adaptations: monomer-level graphs, crystalline input graphs, data-augmented GCNs, attention-based variants, and transfer learning frameworks.

GCNs encode node information and consist of multiple graph convolutional layers passed through a linear regression model, a FCNN for property prediction, or fed through a global average pooling layer.^133–135^ Models can be trained using stochastic gradient descent (SGD), which separates hyperparameter optimization for each property to minimize error.^110,136^ Additionally, architectures can include a boom layer, a fully connected expansion layer introduced to help the model escape local minima and improve convergence^137^ pass the graph level embeddings through a fully connected layers with multisample dropout scheme^138^ to produce the final prediction. Further derivations of GCNs consist of two sequential blocks of gated graph convolutional layers (GCLs). The first block processes the standard molecular graph using gated recurrent units (GRUs) for node updates,^139^ while edge-aware message passing is performed via a learnable transformation matrix computed by a two-layer MLP. After three GCLs, the graph is transformed into a 2-GNN format, where nodes represent atom pairs to capture second-order structural dependencies, as inspired by Grohe et al.^140^ Two additional GCLs are applied to this higher-order graph. The final node embeddings are aggregated using sum pooling and passed through an MLP with ReLU activation and a linear output layer. This two-stage design allows the model to integrate both local and pairwise interactions, enhancing its ability to represent the structural complexity of subunits during pretraining and fine-tuning. Furthermore, GCNs have implemented global nodes to connect every single node in a graph via imaginary edges that participate in message passing to collect global molecular information to retain more information in the overall graph structure, as illustrated in Figure 2.^109^

GCNs provide a simple and flexible framework for polymer property prediction. Across the studies, researchers have adapted the baseline GCN architecture to accommodate data augmentation, crystalline input formats, graph attention, and higher-order message passing. These innovations allow GCN to encode complex topological structure.

### 2.3 GAT and Attention-Based Architectures

Graph Attention Networks (GATs) attempt to learn node embeddings via attention mechanisms.^92,141–143^ Given a graph *G* = (*V*, *E*) with node features *X*, GATs compute node embeddings *h*_*i*_ via attention-weighted aggregation over each node’s neighborhood

6 $$h_{i}=\sigma \left(\sum_{j\in N_{i}\;}\alpha_{\mathrm{ij}}Wx_{j}\right)$$

where *W* is a learnable weight matrix, σ is an activation function, *N*_*i*_ is the neighborhood of node *i*, and α_*ij*_ is the attention coefficient computed as

7 $$\alpha_{\mathrm{ij}}=\frac{\exp\left(\mathrm{LeakyReLU}\left(a^{T}\left[Wx_{i}∥Wx_{j}\right]\right)\right)}{\sum_{k\in N_{i}\;}\exp\left(\mathrm{LeakyReLU}\left(a^{T}\left[Wx_{i}∥Wx_{k}\right]\right)\right)}$$

GATs generate node-level embeddings {*h*_*i*_}, which can be used for downstream tasks such as node classification. For graph-level prediction, a readout function such as a summation, mean, or attention-based pooling over the set of node embeddings before passing them through an output layer (Figure 3).

**Figure 3:**
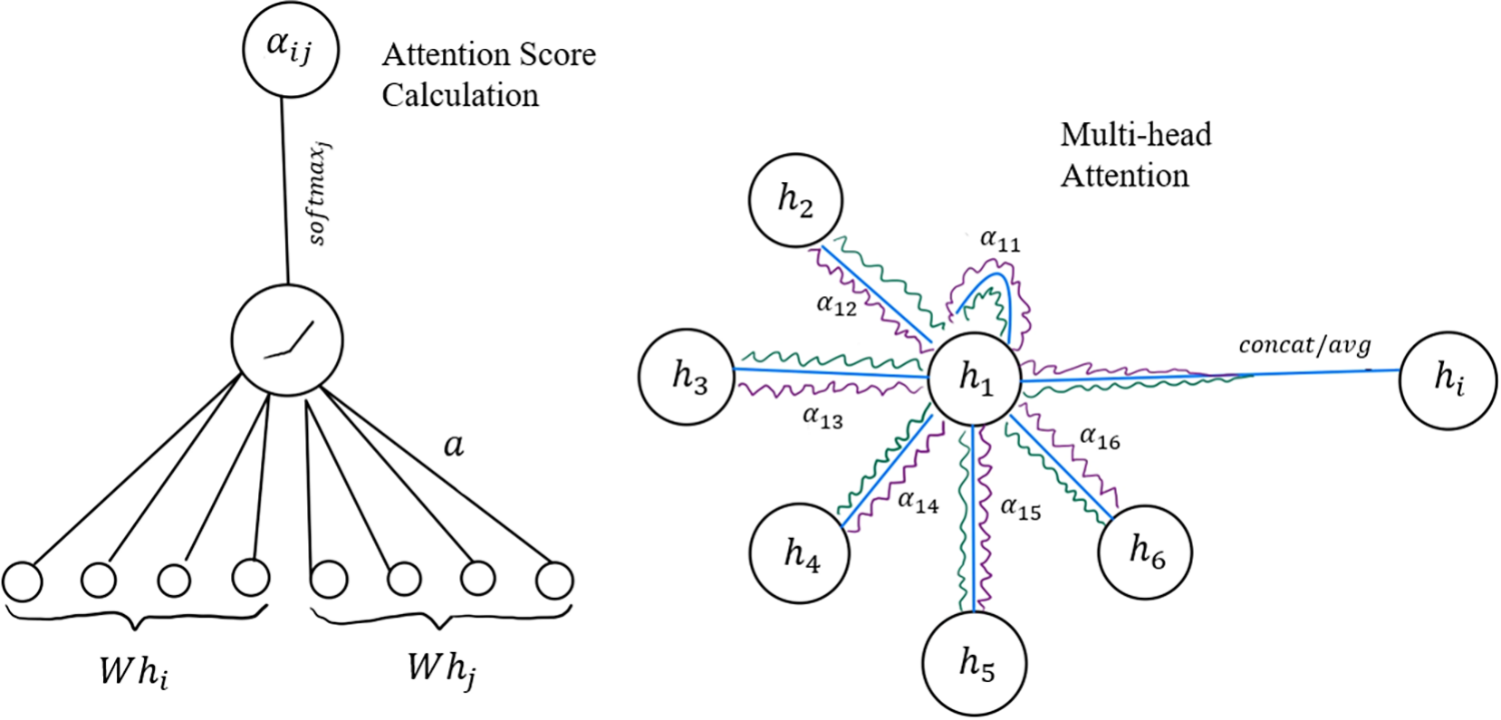
An illustration of GAT operations. The left portion of the diagram shows how attention coefficients α_*ij*_ are computed: the transformed node features *Wh*_*i*_ and *Wh*_*j*_ are concatenated and passed through a shared attention mechanism parametrized by the vector *a*, followed by a LeakyReLU nonlinearity and a softmax normalization over all neighbors *j* ∈ *N*_*i*_. The resulting coefficient α_*ij*_ encodes the learned importance of neighbor *j* when updating node *i*. The right portion illustrates multihead attention for a specific node (node 1 in the drawing): each neighbor contributes messages through multiple attention heads (depicted by the green, blue and purple lines), producing head-specific weights α_1*j*_^(*k*)^ and transformed messages *W* ^(*k*)^ *h*_*j*_. The index *k* refers to the attention head number, where each head has its own learnable weight matrix *W* ^(*k*)^ and produces its own attention score. These per-head aggregations are combined via concatenation (for intermediate layers) or averaging (for final layers) to yield the updated node embedding *h*_*i*_. The diagram highlights both key components of GATs: attention-based neighborhood weighting and multihead message aggregation.

The attention mechanisms weigh neighbor contribution during message passing. An attention mechanism is a neural-network operation that allows the model to assign different importance weights to inputs (or neighboring nodes, in graph settings) when aggregating information. In the context of GNNs, this means that each node learns to focus more on its most relevant neighbors, improving representation flexibility and interpretability compared with uniform aggregation schemes.

Attention mechanisms have been incorporated in polymer graph models in several ways. One applies a GAT-based encoder coupled with a boosted decision-tree regressor. In this setup, the input is represented as a molecular graph and processed using a multihead graph attention layer.^144^ Multihead attention weights highlight the relative importance of neighboring nodes and refine local feature aggregation. The GAT serves as a feature extractor, the resulting graph-level embedding obtained through standard pooling over the node states is then passed to an XGBoost regressor,^145^ which performs supervised prediction using an ensemble of gradient-boosted trees. This hybrid design leverages the representational capacity of attention-based GNNs while benefiting from the speed and tabular regression accuracy of boosted tree models. Another attention-augmented architecture extend this idea to structured, multi-input graph settings. In this model, each component of the system is represented as a separate graph, and a dedicated GNN encoder processes each graph independently.^111^ The encoders combine a GAT layer with a GraphSAGE layer,^80,146^ followed by a self-attention pooling mechanism that outputs a graph-level embedding for each input graph. Sets of related graphs are pooled to produce fixed-size representations, which are then concatenated with additional sample-level attributes to form a unified embedding. This embedding is processed through a fully connected layer to generate a central representation that feeds into one of several output heads, enabling single-task or multitask configurations. During multitask learning, weighted loss functions balance differences in scale across outputs. These architectures are designed to be permutation-invariant to the ordering of input graphs and robust to missing information, making them suitable for screening large combinatorial design spaces.

Although fewer in number, GAT-based architectures demonstrate strong potential for polymer property prediction through adaptive, attention-driven message passing. These models offer dynamic weighting of neighboring features, which enhances representational capacity in chemically diverse and topologically complex graphs. Both GATBoost and POLYMERGNN illustrate how attention mechanisms improve generalization in polymer discovery workflows.

## 3 Data Sets

Polymer data resources range from traditional handbooks to modern ML-oriented databases. Sources such as PolyInfo and Polymer Handbook have validated properties in thousands of polymers, but are not inherently ML readable or designed for the metadata integration required for GNNs.^152–154^ Recent initiatives such as PI1M and the community-driven CRIPT present structured data sets for predictive models.^155,156^ These efforts are a step forward; however the scale and chemical diversity of polymer data sets remains a critical challenge for GNN applications. ML models require a large variety and size of training data.^63,157^ The need for diverse chemistries, accurate structure–property mappings and standardized experimental data further compounds these challenges. Despite the gradual increase in resources, both data scarcity and complexity in data mining in heterogeneous data sets such as OMol25 remain critical bottlenecks for applying GNNs in polymer informatics.^158^

The table below highlights some of the databases available that are used for ML models. First there is a lot of literature that contains information on polymers but here we only highlight two. There is an extensive list of books that contains polymer information, but many are difficult to mine. The Handbook of Polymers contains polymer data on major plastics and other branches in the chemical industry. The data needs to be mined from the book, which contains over 200 types of polymers for research and development.^100^ The Polymer Data Handbook is another book that contains 217 polymers across many modern polymer applications.^159^

Online resources provide easier access to search and find polymer structure-properties, but some are more built for ML models than others. Here we note some of the most common data sets. PubChem is a free resource for searching molecular structures and properties with literature citations. A portion of their data set includes polymers that can be found using their specific names.^98^ MatWeb is another searchable database containing material properties that include data sheets for polymers such as polyester, polycarbonate, polyethelene, and polypropylene. The properties need to be extracted from the platform and reformatted to fit the needs of ML models.^99^ RadonPy is an open source polymer database, developed to process all-atom MD simulations, and 15 properties were calculated for over 1000 amorphous polymers and this data is freely available and in a ML readable form.^160^ PolyInfo provides data for polymeric material design where most of their information is sources from literature on polymers. Their data set contains over 20,000 polymer samples spanning 100 different properties in thermal electrical and mechanical properties across diverse numbers of homopolymers, copolymers, and literature data. Their data set allows for polymer searches, and is free to access but prohibits mass downloading of data.^152^ Polymers: A Property Database provides data for properties and manufacturing processes for over 1000 synthetic and natural polymers. It is a book that requires data mining before implementation in ML studies.^161^ P1IM is a freely accessible benchmark data set containing over 1 million polymers for ML researchers, the model is trained from 12,000 polymers collected from PolyInfo and then trained on a ML generative model for a larger, hypothetical polymer data set to serve as a large benchmark for ML models.^155^ A Polymer Data set for Property Prediction is mainly focused on polyesters, containing 1073 polymers developed from first principle calculations.^162^ CRIPT, the Community Resource for Innovation in Polymer Technology, is a new but growing platform built to easily store and search for polymer materials based on their chemistry and capture relationships between the materials, processes, and data to be used for use in machine learning.^156^ It is a community driven effort for polymeric materials accessibility and designed to be open access for all ML researchers to allow for easy data sharing, and integration through academia, industry and government.

Although specialized literature references such as the Handbook of Polymers and the Polymer Data Handbook provide detailed resources of polymers and the processes that created them, they require manual extraction. Online resources are more searchable, but many are not directly designed for ML research. Table 2 summarizes existing polymer databases and data resources, highlighting the current landscape of available data sets. However, the development of polymer data sets goes beyond open access and format. Polymers are structurally complex, and their representation can differ across platforms which makes cross comparisons difficult. Another issue is imbalance in polymer data sets commonly including industrial polymers, and novel polymers making up a very small portion of the data sets. This leads to an under representations of the diversity needed in a large data set for effective ML learning. Additionally, experimental conditions and property measurements are not standardized in most instances, which can lead to inconsistencies in ML models, and a lack of standardization in validation of data sets can also lead to that same challenge. In order to take the next steps in ML models for polymers, much larger, accessible, and standardized models are needed.

**Figure 4:**
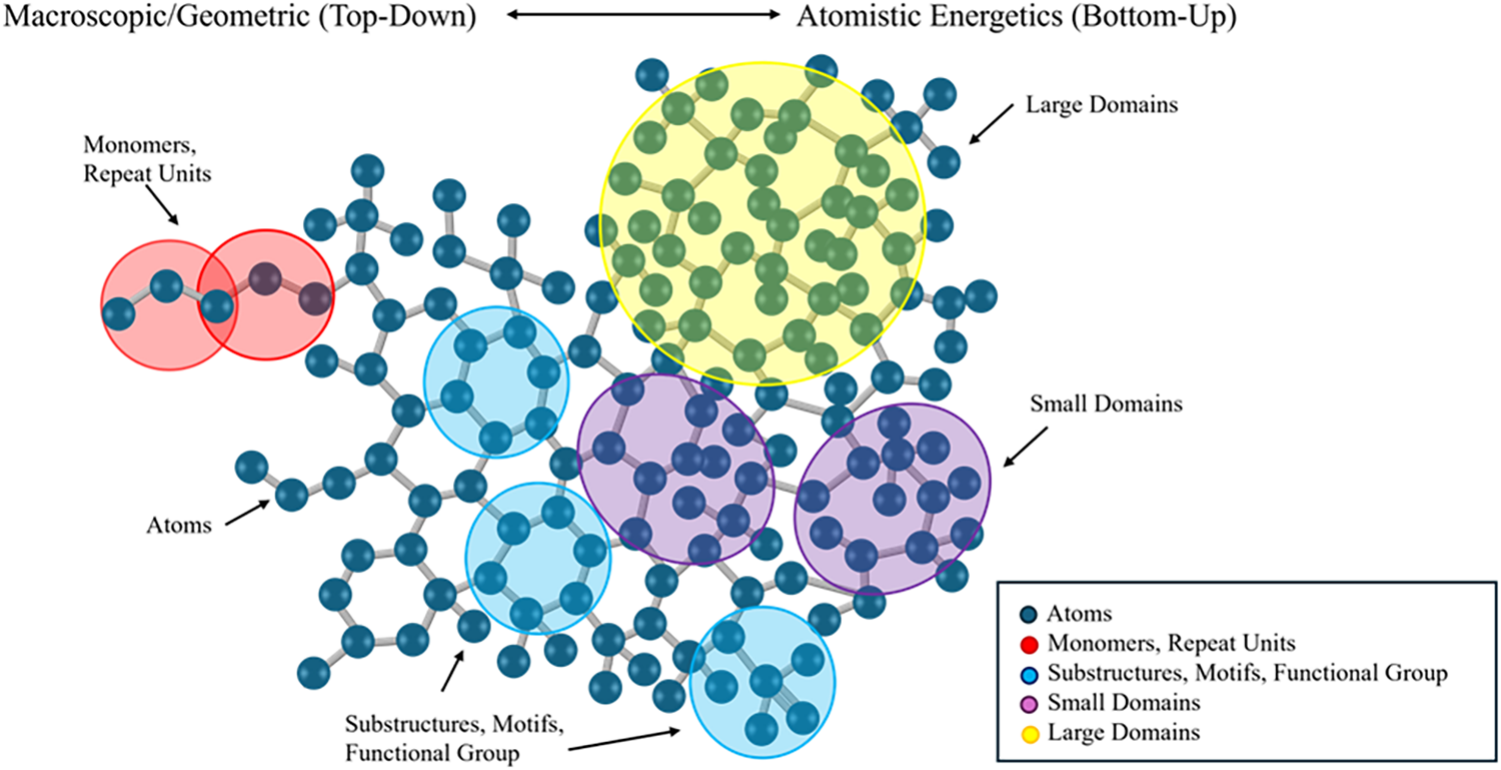
Conceptual depiction of CG in polymer informatics. The top bar represents the (abstract) spectrum of CG strategies from macroscopic, geometry-based top-down strategies to atomistic, energetics-based bottom-up approaches. The molecular structure is overlaid with color-coded regions representing multiple levels of abstraction: atoms (gray), monomer/repeat units (red), substructures, functional groups or motifs (blue), small domains (purple), and large domains (yellow). This hierarchy also illustrates the conceptual transition from atomistic to increasingly coarse-grained graph representations, by grouping chemically or structurally important substructures so that scalable and interpretable ML pipelines are achievable.

**Table 2:** Polymer Databases and Data Resources

database	types of polymers	size/scope
Handbook of Polymers^100^	polymers used by plastics, electronics, and medical fields	over 200 different types of polymers
Polymer Data Handbook^159^	data on physical properties of polymers	217 polymers
PubChem^98^	wide range of polymers and properties	millions of chemical structures, only a portion are polymers
MatWeb^99^	wide range of material properties, including polymers	185,000 materials
RadonPy^160^	15 properties	over 1000 amorphous polymers
PolyInfo^152^	various data for polymeric material design	over 20,000 polymers
Polymers: A Property Database^161^	conducting polymers, hydrogels, nanopolymers and biomaterials	over 1000 polymers
PI1M^155^	polymers for tasks in density, glass transition temperature, melting temperature, and dielectric constants	1 million generated polymers
Polymer Data set for Property Prediction^162^	for the design of high dielectric constant polymers	1073 polymers
CRIPT^156^	polymer data structures stored as graphs containing metadata on properties	size and scope under development
Various small data sets^162–167^	atomization energy, bandgap, dielectric constant, unit cell volume	various sizes

## 4 Coarse-Graining in Polymer Informatics

The direct use of GNN in small molecular systems, where nodes represent atoms and edges bonds have been widely utilized.^79,168,169^ In polymer GNN applications, however, CG is introduced primarily as a graph-representation strategy to control graph size, information content, and learning scalability, rather than as a general molecular-simulation technique (see Figure 4). However, for large systems such as polymers, grouping atoms into higher-level pseudocomponents becomes necessary. In general, CG models in molecular science simplify the representation of complex systems by reducing the level of atomistic detail while retaining critical physical properties, a concept that maps naturally onto the abstraction of nodes and edges in polymer GNN graphs.^170–174^ These models achieve this by grouping atoms into larger pseudoatoms or beads to reduce the number of degrees of freedom.^175–177^ CG enables simulations of larger systems over longer time scales and is computationally more effective overall compared to fully atomistic approaches.^174,178^ This scalability is beneficial for polymer systems in studying mesoscale effects such as polymer diffusion, viscoelasticity, and entanglement.^105,179,180^ However, an inherent limitation of CG models is the loss of detailed atomistic information and the need for careful model parametrization.^102,180,181^ From a GNN perspective, these trade-offs directly determine which chemical, topological, or mesoscale features can be learned at a given graph granularity, and which fine-scale interactions are necessarily discarded. Bead size, mapping of atomistic details and potential energy function accuracy also dictate the accuracy and reliability of the CG model.^103,182,183^ Despite such limitations, CG methods are useful tools for GNNs in property prediction with scalable input representations maintaining useful structural and dynamic information.^184,185^ The integration of CG models with GNNs offers a connection between molecular simulations and real polymer behavior, enabling efficient, structure-aware learning at scale. This is a promising field for polymer informatics, especially in applications in which high-throughput screening and long-time scale behavior are needed.

In polymer GNN literature, hierarchical graph representations directly parallel classical CG concepts, where graph nodes function as CG beads (e.g., monomers, repeat units, substructures, etc.), and graph edges encode the connectivity or interaction topology. The following GNN architectures implement CG in different ways, for example monomers as node graphs, bond centered subgraphs, and repeat unit graphs each correspond to specific CG choices about how atoms are grouped and which interactions are retained. Mohapatra et al.^107^ introduced a CG representation where each monomer was depicted as a node and chemical bonds as edges such that similarity computation, supervised learning, and interpretability could be achieved for glycans and polymers. The approach was effectively used in classification and regression tasks with GNN models in both biological and synthetic systems, with accuracy only slightly lower than all-atom models. Wang et al.^108^ employed a bond-centered subgraph GNN (sGNN), which encoded internal coordinates to describe local environments and outperformed conventional force field methods for large organic molecules. Zhang et al.^106^ introduced the Polymer Unit Graph (PU Graph), which is used for polymer repeat units identified from SMILES using PURS. The model was predictive with accuracy and were interpretable for structure–property relationships. They collectively show CG based GNNs enhance scalability, domain-awareness, and explainability benefits.

There are two major bottlenecks that limit the full integration and application of CG models into polymer GNN pipelines. The first is the challenge of selecting an optimal CG resolution across diverse and complex polymer systems. The accuracy of a CG model depends on how atomic structures are mapped to CG beads, and how this mapping affects the polymer’s structural and dynamical behavior.^174,186^ For some polymers, CG at the monomer scale may be sufficient, while others with complex groups, stereochemical constraints, or cross-linking architectures may require nuanced or hierarchical resolutions CG strategies. CG models are typically tailored to specific polymer chemistries, architectures, or targeted property spaces, model selection must be system dependent and often empirical. The second bottleneck is the lack of robust, high-quality databases at intermediate (mesoscale) resolutions.^187^ While databases exist for macroscopic properties such as mechanical or optical behavior, and atomistic data can be obtained through high-fidelity quantum methods like density functional theory (DFT),^188^ mesoscopic data sets remain sparse.^189^ A comprehensive mesoscale data set would ideally include the polymer chemistry, processing conditions, and environmental variables. However, many available data sets lack reproducibility due to inconsistencies in concentration, thermal, or experimental methodology. Consequently, training and validating CG based models remains difficult due to irregular, incomplete, or underrepresented data. To address this gap experimental data reflecting chemical diversity and process history is essential to extend CG models into broader ML-driven polymer design.

Table 3 provides simply contextual background and terminology, rather than a comprehensive survey, of CG methods, categorized into top-down, bottom-up, and hybrid approaches. Top-down approaches derive coarse-grained potentials by fitting to experimental or macroscopic observables, prioritizing thermodynamic consistency and scalability.^190^ Bottom-up approaches, in contrast, systematically reduce atomistic models by preserving microscopic forces or distributions, offering higher accuracy in reproducing molecular behavior at finer resolution.^191^ Hybrid methods combine both philosophies-retaining atomistic detail in key regions while coarsening others, or integrating field-based and particle-based techniques.^192,193^ While this is not exhaustive, this highlights classic methods, alongside newer ML techniques. These methods vary in their derivation of CG potentials or learned representations and differ in their suitability for modeling goals.

**Table 3:** Overview of Representative CG Models

CG model name	approach type	key features	references
Dissipative Particle Dynamics (DPD)	Top-down	particle-based CG method with soft forces and momentum conservation for mesoscale soft matter	^194–197^
Martini Model	Top-down	4-to-1 (variable) CG model tuned to reproduce experimental thermodynamic data	^198–200^
Self-Consistent Field Theory (SCFT)	Top-down	field-theoretic simulation framework for polymer phase behavior	^201,202^
TraPPE-CG	Top-down	typically for alkanes, parameters fitted to vapor–liquid coexistence	^203^
UNRES	Top-down	united-residue CG force field for protein structure and folding simulations	^204,205^
Multiscale CG (MS-CG)	Bottom-up	derives CG potentials by matching atomistic forces (force-matching)	^172^
Iterative Boltzmann Inversion (IBI)	Bottom-up	iteratively updates CG potentials to match atomistic pair distributions	^206,207^
Single-Chain Boltzmann Inversion	Bottom-up	uses ab initio torsions and intrachain conformational sampling	^208^
United Atom/Blobs	Bottom-up	groups atoms into beads; bonded and nonbonded interactions derived from atomistic structure	^209–211^
Slip-Spring Model	Bottom-up	represents entanglement effects via slip-link dynamics between chains	^212–214^
Bead–Rod Models	Bottom-up	captures chain stiffness using rigid or semiflexible rod-like segments	^215^
Kremer-Grest Model	Bottom-up	idealized bead–spring model for simulating entangled polymer melts	^216–219^
Adaptive Resolution Scheme (AdResS)	Hybrid	couples atomistic and coarse-grained regions dynamically in one simulation box	^220^
SIRAH	Hybrid	CG biomolecular force field combining atomistic mapping with empirical tuning with multiscaling available	^221^
Rosetta	Hybrid	fragment-based modeling using statistical potentials and structural bioinformatics data	^175,222,223^

## 5 Applications and Implementations

Generalizing the architectural frameworks introduced in Section 2, this section reviews the applications of the models for a variety of polymer property prediction tasks. We organize the discussion by target property domains, such as electronic, thermal, and mechanical behavior, to emphasize how various GNN styles relate to specific challenges for polymer modeling.

GNNs have been adapted to a wide variety of polymer property prediction tasks, ranging from electronic and optical properties to thermal, transport, mechanical, and some specialized chemical behaviors. In this section, these architectures are reviewed with a property-oriented focus, organized by application field rather than type of GNN. Although MPNN-based architectures still lead in most type of properties, especially in electronic and thermodynamic tasks, GCN- and GAT-based models have demonstrated competitive or even superior performance in some scenarios. Data set sizes vary widely from a few hundred to tens of thousands of polymers, with sparsity issues of experimental data typically being mitigated using augmentation, transfer learning, or multitask modeling. Model quality is assessed via typical metrics such as RMSE, MAE, or *R* ^2^; architectural variants detailed earlier Section 2 are briefly described here when relevant.

This next subsection provides an overview of the tasks and predictive accuracy of the GNN approaches.

### 5.1 Electronic and Optical Property Prediction

GNNs have been widely applied to predict polymer electronic and optical properties, including carrier mobility, orbital energies, bandgaps, and dielectric constants. This subsection summarizes key contributions based on model architecture, graph representation, and property type. While most models rely on MPNN backbones, several variants incorporate CG representations, periodic graphs, or multitask learning strategies to better capture polymer structure–function relationships.

Zhang et al. proposed the PU-gn-exp model, a MPNN architecture designed for interpretable predictions of optoelectronic properties in organic semiconductors (OSC).^106^ The model replaces atomistic input graphs with polymer-unit (PU) graphs derived from repeat unit parsing. Each PU is encoded as a node, and edges capture interunit connectivity and topology. A gn-exp message-passing network is used with Layerwise Relevance Propagation (LRP)^224^ to enable structure-attribution analysis. The model was trained on a data set of 697 polymers with SMILES inputs and DFT-calculated HOMO, LUMO, and carrier mobility values. PU-gn-exp achieved 81.96 and 88.20% classification accuracy for hole and electron mobility, respectively, while reducing training time by 98% compared to atom-level MPNNs.^91^ This work highlights the interpretability and efficiency benefits of polymer-unit representations.

Aldeghi and Coley evaluated the wD-MPNN model on predicting ionization potential (IP) and electron affinity (EA) on a computationally generated test set of 42,966 copolymers.^121^ The data set used to probe chemical space previously explored by Bai et al.^225^ involved alterations of monomer composition, chain architecture (alternating, random, block), and stoichiometry (1:1, 1:3, 3:1). The EA and IP values were initially predicted using combined extended tight binding (xTB) and DFT calculations, with Boltzmann averaging over eight conformers and several sequences, and latterly calibrated against DFT (B3LYP/DZP) computations.^226–230^ Benchmarked against were Chemprop’s D-MPNN^231^ with disconnected monomer graphs, and fully connected neural networks and RF on ECFP fingerprints.^232,233^ The wD-MPNN with chain architecture via weighted edges and stoichiometry via scaled atomic contributions significantly surpassed these baselines, achieving very low RMSE (0.03 eV) and nearly perfect *R* ^2^ = 1.00 on random splits. On a challenging monomer identity decomposition, it also performed superior with RMSEs of 0.10 eV for EA and 0.09 eV for IP. An ablation study pointed out architectural and stoichiometric encodings as being crucial to model correctness. Moreover, using their model to predict diblock copolymer phase behavior from Arora et al.^50^ experiment data, they achieved a precision-recall curve (PRC) of 0.68, which is higher than that of D-MPNN (0.47) but lower than the RF baseline (0.71). Stoichiometry again proved to be the most important feature.

Zeng et al. applied a GCNN to predict bandgap and dielectric properties from crystal-derived polymer graphs.^110^ They used a database of 1073 polymers compiled from the Polymergenome Project,^162,234,235^ including experimentally synthesized polymers, COD structures, and computationally generated polymers. CIF file structural data included input polymer graphs for the GCNNs. The dielectric constant prediction model had a small mean absolute error (MAE) of 0.24 and performed better than existing Gaussian process regression benchmarks.^235^ Bandgap prediction resulted in slightly greater MAE of 0.41 eV, owing to limited high-bandgap polymers. Notably, bandgap MAE decreased systematically with increasing data set size, demonstrating GCNN scalability. Tests against standard ML baselines (Kernel Ridge Regression, RF, Gradient Boosting, and baseline neural networks) from Matminer-generated descriptors^236^ confirmed GCNN’s superior performance for both properties.

Gurnani et al. investigated the multitask polyGNN, a multitask MPNN that operates on periodic repeat unit graphs for electronic and optical properties.^126^ Their data set was large and included polymer crystal and isolated chain band gaps, ionization energies, electron affinities, experimental and computed refractive indices, and frequency-dependent dielectric constants taken from DFT calculations,^162^ literature,^237,238^ and standard references.^153^ PolyGNN performed better than a PG-MLP baseline trained over Polymer Genome (PG) fingerprints at every point of comparison,^235,239^ specifically achieving smaller RMSE values for electronic properties such as Eg (0.445 eV) and EA (0.380 eV). The multitask setup preserved predictive power even in low-data scenarios, though performance limitations were uncovered for certain low-data dielectric tasks, perhaps owing to limited representation of larger structural motifs and potential over smoothing after only a few message-passing iterations.^240,241^

St. John et al. developed an MPNN model to efficiently screen for organic photovoltaic (OPV) materials and forecast optoelectronic properties directly from 2D molecular connectivity without requiring computational expense of 3D DFT geometry optimization.^127^ Their data set contained approximately 91,000 unique OPV-related molecules with properties computed at both monomer and polymer levels by B3LYP/6–31g­(d).^242^ The single-task MPNN was accurate in prediction with mean absolute errors (MAEs) of 32.1 meV (HOMO) and 27.9 meV (LUMO). Polymer-level predictions were equally accurate for HOMO, LUMO, and optical properties. Although it suggested faster predictions, no concrete runtime benchmarking was provided by the authors. At scale, this work demonstrated that 2D connectivity is sufficient to capture important electronic properties of interest in OPV design.

Antonuik et al. compared three model architectures (RF on monomer descriptors, monomer-graph MPNN, and periodic polymer graph MPNN) on ten polymer properties including atomization energy, bandgap, and electron affinity from a data set of 15,219 computational and experimental data points.^123^ Their periodic graph MPNN achieved the best performance on eight tasks with an average error reduction of 11% relative to monomer-level MPNNs, and 20% relative to descriptor-based RFs. Notable improvements were observed for atomization energy (29%), chain-level bandgap (19%), dielectric constant (17%), and glass transition temperature (6%). Periodic representation was confirmed to be the critical factor enhancing predictivity, with MPNN-generated descriptors being useful only when employed within RF models. Benchmark comparison with results from Kuenneth et al.^243^ further emphasized periodic MPNN’s state-of-the-art predictive accuracy for some properties.

Overall, GNN-based models have demonstrated strong performance across a range of electronic and optical prediction tasks, particularly using MPNNs with polymer-specific graph inputs. Innovations such as adjacency-free architectures,^169^ PU graphs, periodic edge representations, and multitask learning have enabled accurate property prediction. Table 4 provides a structured within-domain summary of the data sets, representation granularity, evaluation protocols, limitations, and main take-home messages for the electronic and optical property studies discussed above.

**Table 4:** Within-Domain Performance Synthesis for Electronic and Optical Polymer Property Prediction with GNNs

study	task	polymer representation	evaluation context	key takeaway/limitation
Zhang et al.^106^	mobility, HOMO/LUMO	polymer-unit graph	random split; classification	interpretability; parsing-dependent
Aldeghi and Coley^121^	IP/EA	stoichiometry- and architecture-aware monomer graph	random splits	weighted atomic contributions
Zeng et al.^110^	bandgap, dielectric	crystal-derived polymer graph	random split; MAE	structure-limited
Gurnani et al.^126^	electronic, optical, dielectric	periodic repeat-unit graph	RMSE	multitask; low-data dielectric limits
St. John et al.^127^	OPV optoelectronics	2D connectivity graph	MAE	scalable screening; runtime not quantified
Antoniuk et al.^123^	multiproperty electronics	periodic polymer graph	relative error reduction	periodicity dominant

### 5.2 Thermal and Mechanical Properties

GNNs have been widely adopted for predicting thermal and mechanical properties of polymers, including glass transition temperature (*T*_g_), melting temperature (*T*_m_), decomposition temperature (*T*_d_) and elastic modulus. These tasks often rely on tailored input representations, such as monomer-based molecular graphs, periodic graphs, or augmented SMILES strings. GNN architectures in this domain range from standard GCNs and MPNNs to multitask or chemically informed frameworks, consistently improving over descriptor-based models. Common strategies to overcome data scarcity include data augmentation, transfer learning, and multitask learning.

Park et al. used GCN-based models to forecast thermal and mechanical properties of polyamides like glass transition temperature (*T*_g_), melting temperature (*T*_m_), density (ρ), and elastic modulus (*E*).^149^ Their data set, sourced from the PoLyInfo database,^152^ consisted of experimental property data for 1,388 polymers (*T*_g_), 942 (*T*_m_), 390 (ρ), and 306 (*E*). They tested two regression models, linear regression (GCN-LR) and fully connected neural network (GCN-NN), against baselines using ECFP descriptors together with LR and NN regressors. The GCN-NN model greatly surpassed ECFP-NN in every instance, particularly doing very well on properties strongly correlated with backbone rigidity. For instance, GCN-NN achieved an extremely high *R* ^2^ of 0.90 (RMSE: 29.98 K) for *T*_g_ and 0.76 (RMSE: 40.37 K) for *T*_*m*_, outperforming ECFP-NN in both tasks. Density predictions were comparable across models, whereas elastic modulus predictions were influenced by noisy and sparse data. Nonlinear transformation using neural networks was identified as of vital importance to all properties. Lastly, transferability of latent representations learned by GCN was demonstrated by projecting 100,000 unlabeled polymers in the PI1M data set onto the same feature space, with uniform structural features as per polymer stiffness. The authors described reduced predictive power for less highly correlated properties and constraints that came from the use of monomer-level data in their model and absence of stereochemical or 3D information.

Gurnani et al. demonstrated polyGNN, a multitask MPNN, for predicting thermal and mechanical polymer properties like decomposition temperature (*T*_d_), melting temperature (*T*_m_), glass transition temperature (*T*_g_), Young’s modulus, and tensile strength.^126^ Their extensive data set consisted of experimentally measured and computationally derived property values, such as *T*_d_ (2200), *T*_m_ (3275), and *T*_g_ (3690), and mechanical properties such as Young’s modulus (412) and tensile strength (466). The multitask polyGNN improved over the PG-MLP baseline consistently for thermal properties with much lower RMSE values: *T*_d_ (58.7 K vs 59.3 K), *T*_m_ (45.0 K vs 47.2 K), and *T*_g_ (31.7 K vs 34.0 K). For mechanical properties, polyGNN performed well, with RMSE of 0.827 GPa (Young’s modulus) and 23.3 MPa (tensile strength), comparable to PG-MLP. Most importantly, polyGNN was capable of achieving positive *R* ^2^ on low-data tasks where individual-task models would perform poorly, highlighting the benefit of multitask learning with selector vector conditioning in capturing heterogeneous polymer behaviors.

Hu et al. used a GCN model with SMILES-based data augmentation to predict polymer glass transition temperatures (*T*_g_) using augmented SMILES strings to transcend data set limitations.^133^ On two data sets (D300 and D600), they employed SMILES enumeration to augment monomer representations, predictive performance significantly enhanced. For instance, augmentation enhanced D300 predictions from RMSE 19.4 K (*R* ^2^: 0.88) to RMSE 7.4 K (*R* ^2^: 0.97) and D600 predictions from RMSE 31.0 K (*R* ^2^: 0.78) to RMSE 15.5 K (*R* ^2^: 0.94). The GCN outperformed consistently descriptor-based baselines (Random Forest, MLP, SVR) and even MPNN models to some extent, especially at elevated augmentation levels, highlighting the power of data augmentation in polymer informatics.

Qiu et al. combined a GNN model and experimental validation to discover high *T*_g_ polyimides (PIs) for demanding aerospace and display applications.^109^ Eight PIs were prepared, with the model estimating experimental *T*_g_ values (seven estimates within 30C). The model applied Atomic Contribution Maps (ACMs) to interpret structure–property correlations, stating that ether bonds and symmetrical methyl groups were undesirable, but aromatic and rigid motifs enhanced *T*_g_. Screening over 8.2 million PI candidates uncovered numerous promising materials, of which 110 have a melting point over 400 C. They also constructed polyScreen, an accessible predictor that can perform fast *T*_g_ predictions. Even though computationally costly, their work suggests the promise of interpretable deep learning methods in polymer design.

Volgin et al. applied transfer learning to a GCNN pretrained on 6.7 million synthetic polyimide units (Askadskii’s QSPR-derived *T*_g_) to predict experimentally measured *T*_g_ values.^151^ Pretraining significantly reduced prediction errors compared to purely experimental data-trained models (MAE: 22.5 K vs 28.1 K). Conversely, general molecular pretrained models (QM9 data set) performed poorly (MAE: 40.6 K), emphasizing the importance of polymer-specific synthetic data. Their findings measure the “reality gap,” suggesting a minimum of 95% synthetic data content for peak performance, consistent with comparable results across other fields.

Hickey et al. compared a GCN trained on a diverse data set (7,558 polymer *T*_g_ values) and a quantum chemistry (QC)-based regression model trained on a small, detailed data set (83 polymers)—for predicting *T*_g_.^134^ The GCN achieved strong accuracy (RMSE: 38.1C, *R* ^2^: 0.90), capturing broad topological diversity, whereas the QC model (RMSE: 34.5C, *R* ^2^: 0.86) provided greater interpretability at lower data scales.

Qiu et al. constructed Chem-DAGNN, a chemically aware GNN using augmented data sets for high-accuracy *T*_g_ prediction with *R* ^2^ = 0.95 and RMSE = 28.0C.^148^ The model outperformed regular GCNs, transformers, and conventional baselines. Screening more than a million polyimides identified new candidates with experimentally verified *T*_g_ above 450C. ACM interpretability also associated structural motifs with enhanced thermal performance, demonstrating the promise of interpretable, augmented data-driven GNNs for advanced polymer discovery.

POLYMERGNN, a multitask attention-based GNN, demonstrated multitask learning robustness for *T*_g_ and intrinsic viscosity (IV) prediction and outperformed kernel ridge regression and traditional descriptors Coulomb Matrices, Smooth overlap of atomic potentials, persistence images and many-body tensor representations.^244–248^ The integration of molecular weight greatly improved IV prediction accuracy. The model’s capability to accurately conduct virtual screening proves its translational potential to guide polymer design with desirable physical properties.

Li et al.’s GATBoost model combined GAT with XGBoost to predict polymer *T*_g_ and identified important structural motifs influencing thermal properties.^144^ Through massive data set augmentation (25 fold via SMILES enumeration), GATBoost attained significant accuracy gains (RMSE reduced to 2.20C, *R* ^2^ = 0.999), surpassing other graph-based and sequence-based models. This highlighted SMILES augmentation’s broad impact as well as the interpretability of GATBoost in sparse data regimes.

In summary, thermal and mechanical property prediction with GNNs benefits from data augmentation, transfer learning, and multitask architectures. While GCN and MPNN backbones remain common in this domain, chemically aware designs and hybrid architectures (e.g., GATBoost, Chem-DAGNN) have led to both accuracy improvements and enhanced model interpretability. Low data regimes are best handled using augmented inputs or multitask conditioning, and interpretability tools are increasingly leveraged to identify functional motifs for targeted materials design. Table 5 summarizes how representation choices and data strategies govern performance in thermal and mechanical property prediction, complementing the architecture-focused discussion in Section 2.

**Table 5:** Within-Domain Performance Synthesis for Thermal and Mechanical Polymer Property Prediction with GNNs

study	task	polymer representation	evaluation context	key takeaway/limitation
Park et al.^149^	*T*_g_, *T*_m_, ρ, *E*	monomer-level molecular graph	random split; RMSE/*R* ^2^	backbone rigidity captured
Gurnani et al.^126^	*T*_g_, *T*_m_, *T*_d_, modulus, strength	periodic repeat-unit graph	RMSE	multitask learning
Hu et al.^133^	*T*_g_	monomer graph with SMILES augmentation	Random split; RMSE	SMILES augmentation
Qiu et al.^109^	High-*T*_g_ PI discovery	monomer graph	screening and experimental validation	interpretability
Volgin et al.^151^	*T*_g_	monomer graph	transfer vs direct training	synthetic pretraining
Hickey et al.^134^	*T*_g_	monomer graph	random split; RMSE/*R* ^2^	small-scale interpretability
Qiu et al.^148^	high-*T*_g_	chemically aware GNN	random split; RMSE/*R* ^2^	data augmentation
POLYMERGNN^244^	*T*_g_, intrinsic viscosity	attention-based graph	random split; RMSE	multitask coupling
Li et al. (GATBoost)^144^	*T*_g_	SMILES graph	random split; RMSE/*R* ^2^	augmentation boosts accuracy; realism unclear

### 5.3 Transport and Dynamic Properties

GNNs have also been used in predicting transport, diffusion, and dynamic properties in polymer systems, which can include time-dependent simulation, polymer–solvent interaction, or force field modeling. These tasks span thermodynamics, local atomic transitions, and nanoparticle formulation. Models in this domain frequently combine GNNs with domain-based information, such as MD, transfer learning, or physics-informed preprocessing, to address the multiscale nature of polymer systems.

Xie et al. developed a hybrid multitask GNN which was trained on short (5 ns) and long (50 ns) molecular dynamics (MD) simulations for the prediction of ionic conductivity (σ) and diffusivity (*D*_Li_, *D*_TFSI_, *D*_Poly_) in polymer electrolytes.^147^ Their data set was reduced from 53,362 polymer candidates to 876 polymers (5 ns MD) and 117 polymers (50 ns MD). The multitask GNN significantly improved predictions, yielding mean absolute errors (MAEs) of 0.076log_10_(S/cm) on interpolated and 0.182 log_10_(S/cm) on extrapolated cases, considerably outperforming short MD baselines and linear correction. The GNN model also performed better than a random forest trained on Morgan fingerprints and exhibited outstanding generalization to experimental data (31 polymers, external MAE: 0.093–0.120 log_10_(S/cm)). This renders the multitask GNN a reliable and effective alternative to high-throughput virtual screening of lithium-ion battery polymer electrolytes.

Sanchez Medina et al. developed GH-GNN for predicting activity coefficients at infinite dilution Ω_*ij*_^∞^ in polymer solvent systems.^124^ Their data set comprised of a curated collection of Ω_*ij*_^∞^ values sourced from various experimental studies using inverse gas chromatography (IGC), encompassing data from over 48 distinct homopolymers and 150 solvents. Various polymer representations including monomers, repeating units, periodic units, and oligomers were tested to determine the impact on model accuracy. The GH-GNN outperformed an RF model achieving a MAE of 0.15–0.26 and an *R* ^2^ of 0.64–0.92 on the data sets for interpolation and extrapolation. With transfer learning, the GNN model was pretrained on a data set from DECHEMA used in their previous work^249^ containing 40,219 data points of smaller molecular systems. The GH-GNN improved further, reducing MAE by approximately 23.5% for the Mn data set, 13.3% for the Mw data set, and another 13.3% for combined Mn/Mw data set. The transfer-learned GH-GNN achieved MAEs near 0.13 and *R* ^2^ above 0.94 for interpolation and 0.15–0.20 MAE and *R* ^2^ above 0.89 extrapolation. The GH-GNN model was compared to additional thermodynamic models such as UNIFAC-ZM,^250^ Entropic-FV,^251^ and other group-contribution-based methods,^252^ consistently outperforming them in both interpolation and extrapolation tasks across diverse polymer–solvent systems. The model demonstrates its strength in modeling complex polymer–solvent behavior and highlights the advantages of combining GNN architectures with transfer learning for mixture property prediction.

Wang et al. evaluated the sGNN, a subgraph-based GNN, to model intramolecular potential energy surfaces for flexible polymers like PEG and PE.^108^ We note that sGNN is a GNN-based ML interatomic potential (MLIP), which is fundamentally different in purpose and scope from the property-prediction GNNs discussed elsewhere in this section. MLIPs for polymers remain relatively limited compared to property-focused GNNs, and a comprehensive survey lies beyond the scope of this review. The target property for learning was the intramolecular energy contribution obtained from second-order Møller–Plesset perturbation theory (MP2), with all nonbonding interactions subtracted using a separate physics-based model.^253^ The training data were derived from NVT molecular dynamics simulations using OPLS-AA on methyl-capped polyethylene glycol (PEG) and polyethylene (PE). They generated 20,000 conformations per small molecule (10,000 each at 300 and 1000 K) and used 1000 randomly selected 300 K conformations for testing. For larger polymers such as PEG[8], only 1000 conformations at 300 K were generated for testing. The sGNN trained on PEG[2] and PEG[4] achieved root-mean-square errors (RMSE) of 0.020 kcal/mol/atom on PEG[4] and PEG[8], outperforming both the classical OPLS-AA force field and the TensorMol-1.0 ML potential, which yielded RMSEs ranging from 0.054–0.24 kcal/mol/atom. They also tested an ablated version of their model trained without removing nonbonding energy, which led to RMSEs of 0.025 kcal/mol/atom on PEG[4] and 0.047 kcal/mol/atom on PEG[8], demonstrating the importance of accurate nonbonding treatment. A message-passing–free variant (“sGNN-local”) showed slightly higher error (0.024 kcal/mol/atom), confirming the role of nonlocal coupling in bonded interactions.

Kimmig et al. applied a GNN to predict nanoparticle size from polymer structure for a data set containing 3753 nanoparticle formulations.^135^ The data set included a diverse range of methacrylates synthesized via controlled radical polymerization techniques, such as reversible addition–fragmentation chain transfer (RAFT) polymerization, ensuring a broad representation of possible polymer structures and sizes. High-throughput nanoprecipitation methods were used to prepare nanoparticles, and dynamic light scattering (DLS) measurements provided precise particle size data. The data was preprocessed to remove outliers and artifacts. During training, the model achieved a mean absolute percentage error (MAPE) of 5.46% ± 0.65% on the training data and 15.12% ± 5.58% on the test data, indicating robust performance across diverse polymer structures and formulation conditions. The high accuracy in predictions for previously unseen polymers underscores the model’s generalizability and potential utility in nanoparticle formulation optimization, significantly reducing the need for extensive experimental trials.

In summary, transport and dynamic property predictions benefit from hybrid GNN approaches that incorporate domain-specific data. GH-GNN leveraged transfer learning for mixture thermodynamics, while sGNN addressed force-field behavior in polymer systems. Across tasks, GNNs demonstrated strong generalization and adaptability when tailored to the chemical or temporal structure of polymers. Table 6 highlights that reliable transport and dynamic property prediction typically requires hybrid GNN pipelines that integrate simulation, physics-based preprocessing, or transfer learning.

**Table 6:** Within-Domain Performance Synthesis for Transport and Dynamic Polymer Property Prediction with GNNs

study	task	polymer representation	evaluation context	key takeaway/limitation
Xie et al.^147^	ionic conductivity, diffusivity	polymer graph	MAE	MD–GNN hybrid
Sanchez Medina et al.^124^	activity coefficients at infinite dilution	polymer–solvent graphs	MAE/*R* ^2^	transfer learning
Wang et al.^108^	intramolecular energy (MLIP)	subgraph-based polymer graph	conformational RMSE vs force fields	near MP2 accuracy achieved; limited to intramolecular PES
Kimmig et al.^135^	nanoparticle size	polymer graph	train/test MAPE	good generalization across chemistries

### 5.4 Biological Properties

GNNs have also been applied to adjacent macromolecular systems beyond classical synthetic polymers, including glycan classification, antimicrobial activity modeling, and viral binding prediction. We include these examples as a brief outlook to illustrate how polymer-inspired graph representations generalize to structurally modular biological macromolecules, rather than as core polymer informatics case studies. Mohapatra et al. developed a CG GNN framework for two supervised learning tasks: classification of glycans and regression of antimicrobial peptide (AMP) activity, both chosen to reflect biologically and chemically relevant macromolecular properties.^107^ For classification, they curated a data set of 1313 labeled glycans from GlycoBase, spanning immunogenicity and eight taxonomic levels. Each glycan was parsed into a graph where monomers were nodes and linkages were edges, and features were generated using either ECFP fingerprints or one-hot encodings. Five GNN architectures were benchmarked: GCN,^90^ Weave,^254^ MPNN,^91^ GAT,^92^ and AttentiveFP,^143^ all implemented via DGL-LifeSci.^255^ All architectures achieved ROC-AUC > 0.95 for both immunogenicity and taxonomy prediction tasks, with AttentiveFP offering the most stable and interpretable attributions. For AMP regression, they used a curated data set of 15,778 peptides from DBAASP, focusing on minimum inhibitory concentration (MIC) predictions against *E. coli* and S. aureus. The Weave model yielded the best results among tested GNN, their framework matched or outperformed prior methods on four out of eight glycan classification tasks, demonstrating that even with coarse-grained inputs, structured monomer-level graphs can achieve high predictive accuracy.

Burkholz et al. introduced SweetNet, a GCN based model enhanced with a boom layer and designed for glycan species and classification.^150^ The species prediction task was framed as a 581-class classification problem, and SweetNet achieved 44.3% accuracy using a GCN with a boom layer, an improvement of 7.79% over the previous method SweetTalk.^256^ For immunogenicity prediction, the model reached an accuracy of 94.6%, exceeding SweetTalk’s 91.7%. In pathogenicity classification, the GCN with a boom layer achieved 91.9% accuracy, outperforming SweetTalk’s 89.1%. Benchmark comparisons across domain, kingdom, phylum, class, order, family, and genus levels showed consistent improvements with SweetNet, with the largest gains seen in highly granular predictions such as genus (11.09% gain over SweetTalk). In addition to taxonomy and functionality, SweetNet was used to predict viral glycan binding intensities an application relevant to molecular recognition. Using 126,894 glycan-binding measurements from glycan arrays of influenza virus hemagglutinin, the model achieved an MSE of 0.7352, outperforming a motif-count-based fully connected network (MSE = 0.8753) and a SweetTalk-based language model (MSE = 0.8726). They were able to capture nuanced chemical features, correctly prioritizing known sialic acid motifs, showing strong generalization, including to glycans not present in the training set.

Collectively, these examples highlight the conceptual transferability of polymer-aware GNN representations to biological macromolecules. While not the focus of (synthetic) polymer informatics, they motivate cross-domain method development and help contextualize why coarse-grained, monomer-level graphs are effective abstractions for structured macromolecular systems (Table 7).

**Table 7:** Within-Domain Performance Synthesis for Biological and Macromolecular Property Prediction with GNNs

study	task	polymer representation	evaluation context	key takeaway/limitation
Mohapatra et al.^107^	glycan classification	coarse-grained monomer graph	ROC–AUC	CG graphs effective for biology
Burkholz et al.^150^	glycan taxonomy, immunogenicity, binding	monosaccharide graph	MSE	interpretability

GNNs have shown solid performance on a wide range of polymer prediction tasks, particularly in electronic and thermal property domains. Multitask learning, CG representations, and data augmentation have worked best at solving data sparsity and generalizability. Message-passing models work better than traditional fingerprint-based models. Transport and mechanical properties are at times more difficult to predict due to noise, sparsity, and complex dynamic behavior opening up direction for future research. This difficulty may reflect architectural limitations as the GNNs reviewed here operate on static molecular graphs and cannot encode chain dynamics or processing history which strongly governs transport and mechanical behavior in real polymer systems. Across these property domains, clear cross-study patterns emerge. *T*_g_ appears repeatedly as a successful target for GCN- and MPNN-based models because it is a scalar, widely recorded experimental property and is well-captured by 2D monomer or repeat-unit graph inputs. This leads to broadly consistent performance across data sets, regardless of architectural variations. By contrast, properties with higher experimental noise, limited data, or more complex physical dependencies (e.g., mechanical moduli or diffusivities) show greater variability in GNN performance. This indicates that data quality and task definition often dominate over architectural choice. These trends provide context on why certain architectures succeed for specific polymer properties and highlight ongoing bottlenecks in low-data and high-noise regimes.

## 6 Discussion and Outlook

GNNs have demonstrated versatility in predicting polymer properties throughout electronic, thermal, transport, and structural regimes. Apart from prediction ability, some recent studies have added interpretability strategies to GNN models, enabling researchers to move away from black-box performance and disclose structure–property relations. This section combines interpretability methods, common findings derived from them, and overall trends and challenges encountered among the surveyed works. It also outlines developing opportunities for polymer GNNs in model building and real-world materials discovery.

### 6.1 Understanding GNNs: Tools and Insights

Across various works, interpretability techniques were employed to move beyond black-box prediction and provide chemical or structural insight into discovered GNN behavior. Some of these included methods such as LRP, saliency maps, integrated gradients, and atomic contribution maps, along with chemically inspired model architectures to reason predictions in terms of structural motifs, functional groups, or topological properties.^257–260^

Zhang et al.^106^ employed LRP with their PU-gn-exp which substitutes atom-level graphs with more CG based repeat-unit graphs to OSCs. Visual and statistical recognition of polymer substructures most responsible for carrier mobility, HOMO, and LUMO prediction was achievable. The interpretability framework was embedded directly into the PyTorch computation graph using automatic differentiation to render attribution both scalable and chemically interpretable.

Additionally, Hu et al.^133^ employed a GCN on SMILES data augmented for the prediction of *T*_g_ to identify atomic features consistent with known thermodynamic behavior with saliency maps. Rigid backbones and bulky side groups were highlighted as influential, consistent with conventional polymer design insight.

Mohapatra et al.^107^ utilized integrated gradients and input × gradient methods to study glycan immunogenicity. Their monomer-level plots show that chemically relevant motifs such as fucose and xylose consistently gave rise to classification issues. Out of several GNNs, AttentiveFP yielded the most stable and interpretable results, pointing to the significance of architecture choice in attribution stability.

Qiu et al. interpreted that the enhanced GNN had learned chemically meaningful rules like favoring rigid, aromatic, hydrogen-bonding and 3,4’-isomer structures that explain and predict high-*T*_*g*_, heat-resistant polyimides.^148^ Li et al.^144^ then extended this further by combining a GAT and an XGBoost into their GATBoost model, where attention scores distinguished significant subgraphs and the boosting algorithm ordered their importance. This two-stage architecture allowed for explainable structure–property.

Across the literature, graph granularity is strongly coupled to property type: atom-level GNNs for local electronic descriptors (HOMO/LUMO, IP/EA), while monomer- or repeat-unit–level graphs perform for long-range or topology-dependent properties such as *T*_g_, diffusivity, or activity coefficients. Data set size also plays a central role: small experimental data sets (<1000 polymers) typically favor architectures with stronger inductive biases, whereas large computational data sets (>10^4^ polymers) allow more expressive MPNNs to achieve their full representational capacity. Although only a few polymer GNN studies use multitask learning, some patterns emerge. When the predicted properties share strong physical coupling-such as thermal transitions (*T*_g_, *T*_m_, *T*_d_) in polyGNN or correlated ionic transport coefficients models improve accuracy. In contrast, when the targets differ in data quality or underlying physics, as seen in the dielectric subtasks, multitask training provides little benefit or may degrade performance. These results suggest that the choice of which tasks to combine often matters more than the specific architecture. Overall, the literature suggests that successful polymer GNNs emerge when graph granularity, data, and multitask structures match the physics of the problem, sometimes exerting a stronger influence on accuracy than from architectural changes alone.

### 6.2 Coarse-Graining in Graph Design

A commonality among the most transferable and explainable models is that they use chemically informed graph representations that are more advanced than the simple atomic graph. In particular, Zhang et al.’s PU-GNN,^106^ Mohapatra et al.’s glycan graphs,^107^ and Wang et al.’s subgraph-based sGNN^108^ all used CG based graph, grouping atoms into chemically functional subunits or repeat motifs. CG representations facilitate easier explanations by aligning model structure with human intuition and domain-specific abstraction. They also offer benefits in training efficiency, generalization, and visual attribution.

This renewed focus on graph-level CG holds promise for unifying representation conventions in polymer informatics. Atomistic graphs remain dominant in small-molecule ML, but polymers offer an inherent modularity-via repeat units, side chains, block architecture, and stereochemistry-that can be fully realized through customized CG representations. These enable researchers to reason over structure–property relationships at a chemically meaningful scale, and even accelerate high-throughput screening pipelines by reducing input complexity without compromising prediction accuracy.

### 6.3 Outlook: from Prediction to Design

As polymer GNNs continue to mature, their application is trending away from predictive accuracy toward active design, synthesis assistance, and discovery. The studies by Qiu et al.,^148^ Hu et al.,^133^ and Li et al.^144^ illustrate this trend, where understandable models are coupled with synthetic verification or screening platforms. Models now can suggest candidates with high *T*_g_, optimal mobility, or desired dielectric properties.

However, some challenges remain. Interpretability metrics vary across studies, and there is no baseline by which the quality of attribution in polymer GNNs can be judged. Morphological and processing effects are often not addressed by current models, even though it is well understood that these influence on polymer properties. Furthermore, CG graph representations contribute to explainability but continue to suffer from a lack of standardization in their definition, parsing, or encoding. At present, one of the biggest challenges in polymer ML is not just the use of existing data sets and their limitations, but also the intentional development of new ones. Polymers are structurally diverse with properties that depend strongly on experimental conditions, and there is currently little standardization across current sources. More direct efforts are needed, such as those led by CRIPT, to create a more uniform framework for polymer data sets. There is a clear need for a polymer-focused data set, where a task force of researchers can run millions of experiments under controlled conditions to build up a large, publicly available, and validated data set for ML models. This community effort should be transparent in the creation of the data, the maintenance of the data, and should avoid bias toward certain types polymers over others to ensure diversity in data sets which is critical for ML models that require that in their training data set to ensure generalizable models. Properties measured under different conditions should be carefully controlled, and computationally derived values should especially be validated before inclusion. Another challenge, is sustainability, many data sets are created for a singular project and then are not maintained or expanded. Overall, many challenges still remain in curating effective and validated, open source data sets that are maintained, balanced, large, and diverse enough for ML models.

To follow up on this work, this field should in the future:

- Develop **benchmark data sets** that integrate property prediction with attribution labels;
- Promote **modular and hierarchical GNN architectures** supporting hybrid input graphs and dynamic behavior predictions;
- Enable **interpretability** with experimental pipelines, e.g., synthesis constraints and descriptors for applied knowledge extractability.

Overall, polymer GNNs have evolved from accurate black-box models to chemically aware and explainable tools. Merging CG graph representations with attribution techniques not only improves transparency but also enables rational polymer design. With increasingly diverse model architectures and larger data sets, interpretable GNNs, especially those based on CG representations are well poised to become the mainstay instruments in data-driven polymer science.

## 7 Conclusions

GNNs have advanced polymer property prediction with adaptable architectures, scalable training, and the ability to model complex structure–property relationships from graph-based molecular representations directly. Across property domains such as electronic, thermal, transport, and beyond GNNs have shown superiority to traditional descriptors and ML pipelines, particularly when tailored to the unique challenges of polymers, such as chain repetition, stereochemistry, and topological heterogeneity.

This review not only highlights the predictive capabilities of MPNNs, GCNs, and multitask GNN variants, but also the increasing trend for polymer-specific innovation in model architecture. These include the use of periodic graphs, PU abstractions, and chemically pertinent edge features, all of which enhance the expressiveness and physical relevance of graph inputs. Furthermore, the integration of explainability tools such as LRP, saliency maps, and attention-based attribution will drive the field forward toward not just accurate but also chemically meaningful models for applied knowledge extractability.

Another direction for the future is the further development of CG graph representations, connecting high-level structure features and low-level graph connectivity. In the PU-GNNs and glycan modeling frameworks, CG graphs allow models to capture mesoscopic polymer properties such as repeat-unit behavior, block copolymer morphology, or motif-based interactions, while maintaining computational tractability and interpretability. Paired with scalable GNN architectures and hybrid learning paradigms (e.g., multitasking, transfer learning), CG representations can offer a promising path forward.

The success of GNNs in polymer informatics will depend on the integration of domain knowledge, standardized graph encodings, benchmarking data sets, and interpretable learning targets. Building on the innovations and challenges discussed here, future work can unleash the potential for graph-based deep learning to accelerate polymer discovery, design, and development.

## References

[1] Liang Y., Yu L. (2010). Development of semiconducting polymers for solar energy harvesting. Polym. Rev..

[2] Baytekin B., Baytekin H. T., Grzybowski B. A. (2013). Retrieving
and converting energy
from polymers: deployable technologies and emerging concepts. Energy Environ. Sci..

[3] Abdelhamid M. E., O’Mullane A. P., Snook G. A. (2015). Storing energy in plastics: a review
on conducting polymers & their role in electrochemical energy
storage. RSC Adv..

[4] Fan, J. ; Njuguna, J. An introduction to lightweight composite materials and their use in transport structures. In Lightweight Composite Structures in Transport; Elsevier, 2016; pp 3–34.

[5] Singh J., Srivastawa K., Jana S., Dixit C., Ravichandran S. (2024). Advancements
in lightweight materials for aerospace structures: A comprehensive
review. Acceleron Aerosp. J..

[6] Yeo S. J., Oh M. J., Yoo P. J. (2019). Structurally controlled
cellular
architectures for high-performance ultra-lightweight materials. Adv. Mater..

[7] Cui H., Zhao Q., Zhang L., Du X. (2020). Intelligent polymer-based
bioinspired actuators: from monofunction to multifunction. Adv. Intell. Syst..

[8] Weng M., Ding M., Zhou P., Ye Y., Luo Z., Ye X., Guo Q., Chen L. (2023). Multi-functional and integrated actuators
made with bio-inspired cobweb carbon nanotube-polymer composites. Chem. Eng. J..

[9] Lendlein A., Rehahn M., Buchmeiser M. R., Haag R. (2010). Polymers in biomedicine
and electronics. Macromol. Rapid Commun..

[10] Medina H., Child N. (2025). A review of developments in carbon-based nanocomposite electrodes
for noninvasive electroencephalography. Sensors.

[11] Farmer C., Medina H. (2023). Effects of electrostriction
on the bifurcated electro-mechanical
performance of conical dielectric elastomer actuators and sensors. Robotica.

[12] Medina, H. ; Farmer, C. ; Liu, I. Dielectric elastomer-based actuators: a modeling and control review for non-experts. In Actuators; MDPI, 2024; Vol. 13, p 151.

[13] Korn D., Farmer C., Medina H. (2022). A detailed
solution framework for
the out-of-plane displacement of circular dielectric elastomer actuators. Eng. Rep..

[14] Peterson G. I., Choi T.-L. (2020). Cascade polymerizations: recent developments in the
formation of polymer repeat units by cascade reactions. Chem. Sci..

[15] Cao C., Lin Y. (2003). Correlation between
the glass transition temperatures and repeating
unit structure for high molecular weight polymers. J. Chem. Inf. Comput. Sci..

[16] Tezuka Y., Oike H. (2001). Topological polymer
chemistry: systematic classification of nonlinear
polymer topologies. J. Am. Chem. Soc..

[17] Gu Y., Zhao J., Johnson J. A. (2019). A (macro)
molecular-level understanding
of polymer network topology. Trends Chem..

[18] Li Y., Abberton B. C., Kröger M., Liu W. K. (2013). Challenges in multiscale
modeling of polymer dynamics. Polymers.

[19] Schmid F. (2023). Understanding
and modeling polymers: The challenge of multiple scales. ACS Polymers Au.

[20] Jones R. (2015). Density functional
theory: Its origins, rise to prominence, and future. Rev. Mod. Phys..

[21] Parr, R. G. Density-Functional Theory of Atoms and Molecules; Oxford University Press, 1989.

[22] Karplus M., McCammon J. A. (2002). Molecular dynamics simulations of biomolecules. Nat. Struct. Biol..

[23] Hollingsworth S. A., Dror R. O. (2018). Molecular dynamics
simulation for all. Neuron.

[24] Tuckerman M. E., Martyna G. J. (2000). Understanding modern
molecular dynamics: Techniques
and applications. J. Phys. Chem. B.

[25] Bhavikatti, S. Finite Element Analysis. New Age International, 2005.

[26] Kim, N.-H. ; Sankar, B. V. ; Kumar, A. V. Introduction to Finite Element Analysis and Design; John Wiley & Sons, 2018.

[27] Farmer C., Medina H. (2024). Hyperelastics. jl: A julia package for hyperelastic
material modelling with a large collection of models. J. Open Source Softw..

[28] Farmer C., Medina H. (2025). Impact-4ccs: Integrated
modeling and prediction using
ab initio and trained potentials for collision cross sections. J. Comput. Chem..

[29] Medina H., Farmer C. (2024). Current challenges in monitoring low contaminant levels
of per-and polyfluoroalkyl substances in water matrices in the field. Toxics.

[30] Farmer C., Medina H. (2025). Universal interatomic potentials with dft for understanding
orbital localization in polydimethylsiloxane-amorphous silica nanocomposites. ACS Omega.

[31] McCormick T. M., Bridges C. R., Carrera E. I., DiCarmine P. M., Gibson G. L., Hollinger J., Kozycz L. M., Seferos D. S. (2013). Conjugated
polymers: Evaluating dft methods for more accurate orbital energy
modeling. Macromolecules.

[32] Adekoya O. C., Adekoya G. J., Sadiku E. R., Hamam Y., Ray S. S. (2022). Application
of dft calculations in designing polymer-based drug delivery systems:
An overview. Pharmaceutics.

[33] Cohen A. J., Mori-Sánchez P., Yang W. (2012). Challenges for density functional
theory. Chem. Rev..

[34] Kohn W., Becke A. D., Parr R. G. (1996). Density
functional theory of electronic
structure. J. Phys. Chem. A.

[35] Dawson W., Degomme A., Stella M., Nakajima T., Ratcliff L. E., Genovese L. (2022). Density functional
theory calculations of large systems:
Interplay between fragments, observables, and computational complexity. Wiley Interdiscip. Rev.: Comput. Mol. Sci..

[36] Bishop M., Kalos M., Frisch H. (1979). Molecular dynamics of polymeric systems. J. Chem. Phys..

[37] Han J., Gee R. H., Boyd R. H. (1994). Glass transition
temperatures of
polymers from molecular dynamics simulations. Macromolecules.

[38] Lau D., Jian W., Yu Z., Hui D. (2018). Nano-engineering of
construction materials using molecular dynamics simulations: Prospects
and challenges. Compos. Part B.

[39] Mackerle J. (2003). Finite element
analysis and simulation of polymersan addendum: a bibliography (1996–2002). Modell. Simul. Mater. Sci. Eng..

[40] Zeng X., Brown L. P., Endruweit A., Matveev M., Long A. C. (2014). Geometrical
modelling of 3d woven reinforcements for polymer composites: Prediction
of fabric permeability and composite mechanical properties. Compos. Part A: Appl. Sci. Manuf..

[41] Alshahrani H. (2020). Characterization
and finite element modeling of coupled properties during polymer composites
forming processes. Mech. Mater..

[42] Thompson A. P., Aktulga H. M., Berger R., Bolintineanu D. S., Brown W. M., Crozier P. S., In’t
Veld P. J., Kohlmeyer A., Moore S. G., Nguyen T. D., Shan R., Stevens M. J., Tranchida J., Trott C., Plimpton S. J. (2022). Lammps-a
flexible simulation tool for particle-based materials modeling at
the atomic, meso, and continuum scales. Comput.
Phys. Commun..

[43] Ratcliff L. E., Mohr S., Huhs G., Deutsch T., Masella M., Genovese L. (2017). Challenges in large scale quantum
mechanical calculations. Wiley Interdiscip.
Rev.: Comput. Mol. Sci..

[44] Fredrickson G. H. (2007). Computational
field theory of polymers: opportunities and challenges. Soft Matter.

[45] Sindu B., Hamaekers J. (2025). Feature-based
prediction of properties of cross-linked
epoxy polymers by molecular dynamics and machine learning techniques. Modell. Simul. Mater. Sci. Eng..

[46] Chew A. K., Afzal M. A. F., Chandrasekaran A., Kamps J. H., Ramakrishnan V. (2024). Designing
the next generation of polymers with machine learning and physics-based
models. Mach. Learn. Sci. Technol..

[47] He J., Rong F. (2025). Prediction of molecularly
imprinted polymer binding affinity based
on graph neural networks. J. Phys.: Conference
Series.

[48] Nagasawa S., Al-Naamani E., Saeki A. (2018). Computer-aided screening of conjugated
polymers for organic solar cell: classification by random forest. J. Phys. Chem. Lett..

[49] Kishino M., Matsumoto K., Kobayashi Y., Taguchi R., Akamatsu N., Shishido A. (2023). Fatigue life
prediction of bending polymer films using
random forest. Int. J. Fatigue.

[50] Arora A., Lin T.-S., Rebello N. J., Av-Ron S. H., Mochigase H., Olsen B. D. (2021). Random forest predictor
for diblock copolymer phase
behavior. ACS Macro Lett..

[51] Malashin I., Tynchenko V., Gantimurov A., Nelyub V., Borodulin A. (2025). Support vector
machines in polymer science: a review. Polymers.

[52] Ziaee H., Hosseini S. M., Sharafpoor A., Fazavi M., Ghiasi M. M., Bahadori A. (2015). Prediction of solubility
of carbon dioxide in different
polymers using support vector machine algorithm. J. Taiwan Inst. Chem. Eng..

[53] Chen F.-C. (2019). Virtual
screening of conjugated polymers for organic photovoltaic devices
using support vector machines and ensemble learning. Int. J. Polym. Sci..

[54] Brereton R. G., Lloyd G. R. (2010). Support vector machines for classification and regression. Analyst.

[55] Youshia J., Ali M. E., Lamprecht A. (2017). Artificial neural network based particle
size prediction of polymeric nanoparticles. Eur. J. Pharm. Biopharm..

[56] Zhang Z., Friedrich K. (2003). Artificial neural networks applied
to polymer composites:
a review. Compos. Sci. Technol..

[57] El
Kadi H. (2006). Modeling the mechanical behavior of fiber-reinforced polymeric composite
materials using artificial neural networksa review. Compos. Struct..

[58] Liu W., Cao C. (2009). Artificial neural network
prediction of glass transition temperature
of polymers. Colloid Polym. Sci..

[59] Chen L., Pilania G., Batra R., Huan T. D., Kim C., Kuenneth C., Ramprasad R. (2021). Polymer informatics: Current status
and critical next steps. Mater. Sci. Eng. R.

[60] Butler K. T., Davies D. W., Cartwright H., Isayev O., Walsh A. (2018). Machine learning
for molecular and materials science. Nature.

[61] Schleder G. R., Padilha A. C. M., Acosta C. M., Costa M., Fazzio A. (2019). From dft to
machine learning: Recent approaches to materials science-a review. J. Chem. Inf. Model..

[62] Sha W., Li Y., Tang S., Tian J., Zhao Y., Guo Y., Zhang W., Zhang X., Lu S., Cao Y.-C., Cheng S. (2021). Machine learning in polymer informatics. InfoMat.

[63] Martin T.
B., Audus D. J. (2023). Emerging
trends in machine learning: a polymer perspective. ACS Polymers Au.

[64] Ramprasad R., Batra R., Pilania G., Mannodi-Kanakkithodi A., Kim C. (2017). Machine learning in materials informatics:
recent applications and
prospects. npj Comput. Mater..

[65] Chen G., Shen Z., Iyer A., Ghumman U. F., Tang S., Bi J., Chen W., Li Y. (2020). Machine-learning-assisted
de novo
design of organic molecules and polymers: opportunities and challenges. Polymers.

[66] Wu, S. ; Yamada, H. ; Hayashi, Y. ; Zamengo, M. ; Yoshida, R. Potentials and challenges of polymer informatics: exploiting machine learning for polymer design, 2020, arXiv:2010.07683. arXiv.org e-Print archive https://arxiv.org/abs/2010.07683.

[67] Kumar J. N., Li Q., Jun Y. (2019). Challenges and opportunities
of polymer design with
machine learning and high throughput experimentation. MRS Commun..

[68] Wieder O., Kohlbacher S., Kuenemann M., Garon A., Ducrot P., Seidel T., Langer T. (2020). A compact review of molecular property
prediction with graph neural networks. Drug
Discovery Today: Technol..

[69] Wu Z., Pan S., Chen F., Long G., Zhang C., Philip S. Y. (2021). A comprehensive
survey on graph neural networks. IEEE Trans.
Neural Netw. Learn. Syst..

[70] Gao Q., Dukker T., Schweidtmann A. M., Weber J. M. (2024). Self-supervised
graph neural networks for polymer property prediction. Mol. Syst. Des. Eng..

[71] Inae, E. ; Liu, Y. ; Zhu, Y. ; Xu, J. ; Liu, G. ; Zhang, R. ; Luo, T. ; Jiang, M. Modeling Polymers with Neural Networks; American Chemical Society, 2025.

[72] Gurney, K. An Introduction to Neural Networks; CRC Press, 2018.

[73] Haykin S., Network N. (2004). A comprehensive foundation. Neural
Netw..

[74] LeCun Y., Bottou L., Bengio Y., Haffner P. (1998). Gradient-based learning
applied to document recognition. Proc. IEEE.

[75] Li Z., Liu F., Yang W., Peng S., Zhou J. (2022). A survey of convolutional
neural networks: analysis, applications, and prospects. IEEE Trans. Neural Netw. Learn. Syst..

[76] Gu J., Wang Z., Kuen J., Ma L., Shahroudy A., Shuai B., Liu T., Wang X., Wang G., Cai J., Chen T. (2018). Recent advances in
convolutional neural networks. Pattern Recognit..

[77] Ge W., De Silva R., Fan Y., Sisson S. A., Stenzel M. H. (2025). Machine
learning in polymer research. Adv. Mater..

[78] Platonov, O. ; Kuznedelev, D. ; Diskin, M. ; Babenko, A. ; Prokhorenkova, L. A critical look at the evaluation of gnns under heterophily: Are we really making progress? 2023, arXiv:2302.11640. arXiv.org e-Print archive https://arxiv.org/abs/2302.11640.

[79] Wang, Y. ; Li, Z. ; Barati Farimani, A. Graph neural networks for molecules. In Machine Learning in Molecular Sciences; Springer, 2023; pp 21–66.

[80] Hamilton, W. ; Ying, Z. ; Leskovec, J. Inductive representation learning on large graphs. In NIPS’17: Proceedings of the 31st International Conference on Neural Information Processing Systems; Department of Computer Science: Stanford, CA, 2017; pp 1025–1035.

[81] Xu, K. ; Hu, W. ; Leskovec, J. ; Jegelka, S. How powerful are graph neural networks? 2018, arXiv:1810.00826. arXiv.org e-Print archive https://arxiv.org/abs/1810.00826.

[82] Sharma K., Lee Y.-C., Nambi S., Salian A., Shah S., Kim S.-W., Kumar S. (2024). A survey of graph neural networks
for social recommender systems. ACM Computing
Surveys.

[83] Kumar S., Mallik A., Khetarpal A., Panda B. S. (2022). Influence maximization
in social networks using graph embedding and graph neural network. Informat. Sci..

[84] Zhang X.-M., Liang L., Liu L., Tang M.-J. (2021). Graph neural
networks
and their current applications in bioinformatics. Front. Genetics.

[85] Malla, A. M. ; Banka, A. A. A systematic review of deep graph neural networks: Challenges, classification, architectures, applications & potential utility in bioinformatics, 2023, arXiv:2311.02127. arXiv.org e-Print archive https://arxiv.org/abs/2311.02127.

[86] Zheng, P. ; Wang, S. ; Wang, X. ; Zeng, X. Artificial intelligence in bioinformatics and drug repurposing: methods and applications. In Frontiers Research Topics, 2022. 10.3389/fgene.2022.870795 PMC8969764 35368698

[87] Reiser P., Neubert M., Eberhard A., Torresi L., Zhou C., Shao C., Metni H., van Hoesel C., Schopmans H., Sommer T., Friederich P. (2022). Graph neural
networks for materials science and chemistry. Commun. Mater..

[88] Fung V., Zhang J., Juarez E., Sumpter B. G. (2021). Benchmarking graph
neural networks for materials chemistry. npj
Computat. Mater..

[89] Maurizi M., Gao C., Berto F. (2022). Predicting
stress, strain and deformation fields in
materials and structures with graph neural networks. Sci. Rep..

[90] Kipf, T. N. ; Welling, M. Semi-supervised classification with graph convolutional networks, 2016, arXiv:1609.02907. arXiv.org e-Print archive https://arxiv.org/abs/1609.02907.

[91] Gilmer, J. ; Schoenholz, S. S. ; Riley, P. F. ; Vinyals, O. ; Dahl, G. E. Neural message passing for quantum chemistry. In International Conference on Machine Learning; PMLR, 2017; pp 1263–1272.

[92] Veličković, P. ; Cucurull, G. ; Casanova, A. ; Romero, A. ; Lio, P. ; Bengio, Y. “Graph attention networks, 2017, arXiv:1710.10903. arXiv.org e-Print archive https://arxiv.org/abs/1710.10903.

[93] Liu, G. ; Inae, E. ; Jiang, M. Deep learning for polymer property prediction. In Deep Learning for Polymer Discovery: Foundation and Advances; Springer, 2025; pp 17–35.

[94] Dong C., Li D., Liu J. (2024). Glass transition
temperature prediction of polymers
via graph reinforcement learning. Langmuir.

[95] Wang X., Liao L., Huang C., Wu X. (2026). Prediction of mechanical
properties of cross-linked polymer interface by graph convolution
network. Acta Mechanica Sinica.

[96] Safari, H. ; Bavarian, M. Enhancing polymer reaction engineering through the power of machine learning. In Systems and Control Transactions, 2024; p 157792.

[97] Liao H.-C., Lin Y.-H., Peng C.-H., Li Y.-P. (2025). Directed message
passing neural networks for accurate prediction of polymer-solvent
interaction parameters. ACS Engineering Au.

[98] Kim S., Chen J., Cheng T., Gindulyte A., He J., He S., Li Q., Shoemaker B. A., Thiessen P. A., Yu B. (2023). Pubchem
2023 update. Nucleic Acids Res..

[99] Gao Z.-Y., Liu G.-Q. (2013). Recent progress of web-enable material database and
a case study of nims and matweb. J. Mater. Eng..

[100] Wypych, G. Handbook of Polymers; Elsevier, 2022.

[101] Patra A., Batra R., Chandrasekaran A., Kim C., Huan T. D., Ramprasad R. (2020). A multi-fidelity information-fusion
approach to machine learn and predict polymer bandgap. Comput. Mater. Sci..

[102] Dhamankar S., Webb M. A. (2021). Chemically specific
coarse-graining
of polymers: methods and prospects. J. Polym.
Sci..

[103] Müller-Plathe F. (2002). Coarse-graining in polymer simulation: from the atomistic
to the mesoscopic scale and back. ChemPhysChem.

[104] Harmandaris V. A., Reith D., Van der
Vegt N. F., Kremer K. (2007). Comparison between coarse-graining
models for polymer
systems: Two mapping schemes for polystyrene. Macromol. Chem. Phys..

[105] Padding J. T., Briels W. J. (2011). Systematic coarse-graining
of the
dynamics of entangled polymer melts: the road from chemistry to rheology. J. Phys.: Condens. Matter.

[106] Zhang X., Sheng Y., Liu X., Yang J., Goddard W. A., Ye C., Zhang W. (2024). Polymer-unit
graph: Advancing interpretability in graph neural network machine
learning for organic polymer semiconductor materials. J. Chem. Theory Comput..

[107] Mohapatra S., An J., Gómez-Bombarelli R. (2022). Chemistry-informed
macromolecule graph representation for similarity computation, unsupervised
and supervised learning. Machine Learning: Sci.
Technol..

[108] Wang X., Xu Y., Zheng H., Yu K. (2021). A scalable
graph neural network method for developing an accurate force field
of large flexible organic molecules. J. Phys.
Chem. Lett..

[109] Qiu H., Qiu X., Dai X., Sun Z.-Y. (2023). Design
of polyimides
with targeted glass transition temperature using a graph neural network. J. Mater. Chem. C.

[110] Zeng, M. ; Kumar, J. N. ; Zeng, Z. ; Savitha, R. ; Chandrasekhar, V. R. ; Hippalgaonkar, K. “Graph convolutional neural networks for polymers property prediction, 2018, arXiv:1811.06231. arXiv.org e-Print archive https://arxiv.org/abs/1811.06231.

[111] Queen O., McCarver G. A., Thatigotla S., Abolins B. P., Brown C. L., Maroulas V., Vogiatzis K. D. (2023). Polymer
graph neural networks for multitask property learning. npj Computat. Mater..

[112] Bongini P., Bianchini M., Scarselli F. (2021). Molecular
generative graph neural networks for drug discovery. Neurocomputing.

[113] Scarselli F., Gori M., Tsoi A. C., Hagenbuchner M., Monfardini G. (2009). The graph neural network model. IEEE Transact. Neural Networks.

[114] Dwivedi V. P., Joshi C. K., Luu A. T., Laurent T., Bengio Y., Bresson X. (2023). Benchmarking graph neural networks. J. Machine Learning Res..

[115] Veličković P. (2023). Everything is connected:
Graph neural
networks. Curr. Opin. Struct. Biol..

[116] Jegelka, S. Theory of graph neural networks: Representation and learning. In International Congress of Mathematicians, 2022; pp 1–23.

[117] Liu, Z. ; Zhou, J. Introduction to Graph Neural Networks; Springer Nature, 2022.

[118] Tang M., Li B., Chen H. (2023). Application
of message
passing neural networks for molecular property prediction. Curr. Opin. Struct. Biol..

[119] Hasebe T. (2021). Knowledge-embedded message-passing
neural networks:
improving molecular property prediction with human knowledge. ACS omega.

[120] Zhang H., Lin Y., Li S., Dai M., Zhang Y., Huang L., Pang J., Wu P., Peng J., Tang Z., Ding P., Xiao W., Song N., Dongbo D. (2025). Substructure-enhanced mpnn for polymer
discovery and knowledge: A study in predicting glass transition temperature. Macromolecules.

[121] Aldeghi M., Coley C. W. (2022). A graph representation of molecular
ensembles for polymer property prediction. Chem.
Sci..

[122] Heid E., Greenman K. P., Chung Y., Li S.-C., Graff D. E., Vermeire F. H., Wu H., Green W. H., McGill C. J. (2024). Chemprop:
a machine learning package for chemical property
prediction. J. Chem. Inf. Model..

[123] Antoniuk E. R., Li P., Kailkhura B., Hiszpanski A. M. (2022). Representing polymers as periodic graphs with learned
descriptors for accurate polymer property predictions. J. Chem. Inf. Model..

[124] Sanchez Medina E. I., Kunchapu S., Sundmacher K. (2023). Gibbs-helmholtz
graph neural network for the prediction of activity coefficients of
polymer solutions at infinite dilution. J. Phys.
Chem. A.

[125] Battaglia, P. W. ; Hamrick, J. B. ; Bapst, V. ; Sanchez-Gonzalez, A. ; Zambaldi, V. ; Malinowski, M. ; Tacchetti, A. ; Raposo, D. ; Santoro, A. ; Faulkner, R. ; C, G. ; F, S. ; Ballard, A. ; Justin, G. ; George, D. ; Ashish, V. ; Kelsey, A. ; Charles, N. ; Victoria, L. ; Chris, D. ; Nicolas, H. ; Dann, W. ; Pushmeet, K. ; Matt, B. ; Oriol, V. ; Yujia, L. ; Razvan, P. Relational inductive biases, deep learning, and graph networks, 2018, arXiv:1806.01261. arXiv.org e-Print archive https://arxiv.org/abs/1806.01261.

[126] Gurnani R., Kuenneth C., Toland A., Ramprasad R. (2023). Polymer informatics
at scale with multitask graph neural networks. Chem. Mater..

[127] St John P. C., Phillips C., Kemper T. W., Wilson A. N., Guan Y., Crowley M. F., Nimlos M. R., Larsen R. E. (2019). Message-passing
neural networks for high-throughput polymer screening. J. Chem. Phys..

[128] Li, Y. ; Zemel, R. ; Brockschmidt, M. ; Tarlow, D. Gated graph sequence neural networks. In Proceedings of ICLR16, 2016.

[129] Dey, R. ; Salem, F. M. Gate-variants of gated recurrent unit (gru) neural networks. In 2017 IEEE 60th international midwest symposium on circuits and systems (MWSCAS); IEEE, 2017; pp 1597–1600.

[130] Sun M., Zhao S., Gilvary C., Elemento O., Zhou J., Wang F. (2020). Graph convolutional networks for computational drug development and
discovery. Briefings Bioinf..

[131] Xu X., Zhao X., Wei M., Li Z. (2023). A comprehensive review
of graph convolutional networks: approaches and applications. Electronic Res. Archive.

[132] Zhou J., Cui G., Hu S., Zhang Z., Yang C., Liu Z., Wang L., Li C., Sun M. (2019). Graph neural networks: A review of methods and applications. IEEE Transactions on Pattern Analysis and Machine Intelligence.

[133] Hu J., Li Z., Lin J., Zhang L. (2023). Prediction and interpretability
of glass transition temperature of homopolymers by data-augmented
graph convolutional neural networks. ACS Appl.
Mater. Interfaces.

[134] Hickey K., Feinstein J., Sivaraman G., MacDonell M., Yan E., Matherson C., Coia S., Xu J., Picel K. (2024). Applying machine learning
and quantum chemistry to predict the glass transition temperatures
of polymers. Comput. Mater. Sci..

[135] Kimmig J., Schuett T., Vollrath A., Zechel S., Schubert U. S. (2021). Prediction of nanoparticle sizes
for arbitrary methacrylates
using artificial neuronal networks. Adv. Sci..

[136] Tian Y., Zhang Y., Zhang H. (2023). Recent advances
in
stochastic gradient descent in deep learning. Mathematics.

[137] Merity, S. “Single headed attention rnn: Stop thinking with your head, 2019, arXiv:1911.11423. arXiv.org e-Print archive https://arxiv.org/abs/1911.11423.

[138] Inoue, H. Multi-sample dropout for accelerated training and better generalization, 2019, arXiv:1905.09788. arXiv.org e-Print archive https://arxiv.org/abs/1905.09788.

[139] Cho, K. ; Van Merriënboer, B. ; Bahdanau, D. ; Bengio, Y. On the properties of neural machine translation: Encoder-decoder approaches, 2014, arXiv:1409.1259. arXiv.org e-Print archive https://arxiv.org/abs/1409.1259.

[140] Morris C., Ritzert M., Fey M., Hamilton W. L., Lenssen J. E., Rattan G., Grohe M. (2019). Weisfeiler and leman
go neural: Higher-order graph neural networks. Proceedings of the AAAI Conference on Artificial Intelligence.

[141] Ye X.-b., Guan Q., Luo W., Fang L., Lai Z.-R., Wang J. (2022). Molecular substructure
graph attention
network for molecular property identification in drug discovery. Pattern Recognition.

[142] Vrahatis A. G., Lazaros K., Kotsiantis S. (2024). Graph attention
networks: a comprehensive review of methods and applications. Future Internet.

[143] Xiong Z., Wang D., Liu X., Zhong F., Wan X., Li X., Li Z., Luo X., Chen K., Jiang H., Z M (2020). Pushing the boundaries
of molecular representation for drug discovery
with the graph attention mechanism. J. Med.
Chem..

[144] Li D., Ru Y., Liu J. (2024). Gatboost: Mining graph attention
networks-based important substructures of polymers for a better property
prediction. Mater. Today Commun..

[145] Chen, T. ; Guestrin, C. Xgboost: A scalable tree boosting system. In Proceedings of the 22nd ACM SIGKDD International Conference on Knowledge Discovery and Data Mining, 2016; pp 785–794.

[146] Liu T., Jiang A., Zhou J., Li M., Kwan H. K. (2023). Graphsage-based
dynamic spatial-temporal graph convolutional network for traffic prediction. IEEE Transactions on Intelligent Transportation Systems.

[147] Xie T., France-Lanord A., Wang Y., Lopez J., Stolberg M. A., Hill M., Leverick G. M., Gomez-Bombarelli R., Johnson J. A., Shao-Horn Y., Grossman J. C. (2022). Accelerating amorphous
polymer electrolyte screening by learning to reduce errors in molecular
dynamics simulated properties. Nat. Commun..

[148] Qiu H., Wang J., Qiu X., Dai X., Sun Z.-Y. (2024). Heat-resistant
polymer discovery by utilizing interpretable graph neural network
with small data. Macromolecules.

[149] Park J., Shim Y., Lee F., Rammohan A., Goyal S., Shim M., Jeong C., Kim D. S. (2022). Prediction
and interpretation of polymer properties using the graph convolutional
network. ACS Polymers Au.

[150] Burkholz R., Quackenbush J., Bojar D. (2021). Using graph convolutional
neural networks to learn a representation for glycans. Cell Rep..

[151] Volgin I. V., Batyr P. A., Matseevich A. V., Dobrovskiy A. Y., Andreeva M. V., Nazarychev V. M., Larin S. V., Goikhman M. Y., Vizilter Y. V., Askadskii A. A., Lyulin S. V. (2022). Machine learning with enormous “synthetic”
data sets: Predicting glass transition temperature of polyimides using
graph convolutional neural networks. ACS Omega.

[152] Otsuka, S. ; Kuwajima, I. ; Hosoya, J. ; Xu, Y. ; Yamazaki, M. Polyinfo: Polymer database for polymeric materials design. In 2011 International Conference on Emerging Intelligent Data and Web Technologies; IEEE, 2011; pp 22–29.

[153] Brandrup, J. ; Immergut, E. H. ; Grulke, E. A. ; Abe, A. ; Bloch, D. R. Polymer Handbook; Wiley: New York, 1999; Vol. 89.

[154] Mark, J. E. Physical Properties of Polymers Handbook; Springer, 2007; Vol. 1076.

[155] Ma R., Luo T. (2020). Pi1m: a benchmark database for polymer informatics. J. Chem. Inf. Model..

[156] Walsh, D. J. ; Zou, W. ; Schneider, L. ; Mello, R. ; Deagen, M. E. ; Mysona, J. ; Lin, T.-S. ; de Pablo, J. J. ; Jensen, K. F. ; Audus, D. J. Community resource for innovation in polymer technology (cript): a scalable polymer material data structure, 2023. 10.1021/acscentsci.3c00011 PMC10037456 36968543

[157] Yan C., Li G. (2023). The rise of machine learning in polymer discovery. Advanced Intelligent Systems.

[158] Levine, D. S. ; Shuaibi, M. ; Spotte-Smith, E. W. C. ; Taylor, M. G. ; Hasyim, M. R. ; Michel, K. ; Batatia, I. ; Csányi, G. ; Dzamba, M. ; Eastman, P. The open molecules 2025 (omol25) dataset, evaluations, and models, 2025, arXiv:2505.08762. arXiv.org e-Print archive https://arxiv.org/abs/2505.08762.

[159] Mark, J. E. Polymer Data Handbook, Oxford University Press, New York, NY, 2009.

[160] Hayashi Y., Shiomi J., Morikawa J., Yoshida R. (2022). Radonpy: automated
physical property calculation using all-atom classical molecular dynamics
simulations for polymer informatics. npj Computat.
Mater..

[161] Ellis, B. ; Smith, R. Polymers: A Property Database. CRC Press, 2008.

[162] Huan T. D., Mannodi-Kanakkithodi A., Kim C., Sharma V., Pilania G., Ramprasad R. (2016). A polymer
dataset for accelerated property prediction and design. Scientific data.

[163] Pence, H. E. ; Williams, A. Chemspider: an online chemical information resource, 2010.

[164] Polymer property predictor and database. https://pppdb.uchicago.edu/, 2019–2025.

[165] Campus plastic s database: Computer aided material preselection by uniform standards. https://www.campusplastics.com/, 1988–2025.

[166] Miccio L.
A., Schwartz G. A. (2020). From chemical
structure to quantitative
polymer properties prediction through convolutional neural networks. Polymer.

[167] Xu P., Lu T., Ju L., Tian L., Li M., Lu W. (2021). Machine learning aided design of polymer with targeted
band gap based
on dft computation. J. Phys. Chem. B.

[168] Xiong J., Xiong Z., Chen K., Jiang H., Zheng M. (2021). Graph neural
networks for automated de novo drug design. Drug Discovery Today.

[169] Medina H., Drake R., Farmer C. (2025). Accelerated
prediction
of molecular properties for per-and polyfluoroalkyl substances using
graph neural networks with adjacency-free message passing. Environ. Pollut..

[170] Tozzini V. (2005). Coarse-grained models for proteins. Curr. Opin. Struct. Biol..

[171] Joshi S. Y., Deshmukh S. A. (2021). A review of advancements
in coarse-grained
molecular dynamics simulations. Mol. Simul..

[172] Noid W. G., Liu P., Wang Y., Chu J.-W., Ayton G. S., Izvekov S., Andersen H. C., Voth G. A. (2008). The multiscale
coarse-graining method. ii. numerical implementation for coarse-grained
molecular models. J. Chem. Phys..

[173] Pak A. J., Voth G. A. (2018). Advances in coarse-grained
modeling
of macromolecular complexes. Curr. Opin. Struct.
Biol..

[174] Noid W. G. (2013). Perspective:
Coarse-grained models for biomolecular
systems. J. Chem. Phys..

[175] Kmiecik S., Gront D., Kolinski M., Wieteska L., Dawid A. E., Kolinski A. (2016). Coarse-grained protein
models and
their applications. Chem. Rev..

[176] Ingólfsson H. I., Lopez C. A., Uusitalo J. J., de Jong D. H., Gopal S. M., Periole X., Marrink S. J. (2014). The power of coarse
graining in biomolecular simulations. Wiley
Interdiscip. Rev.: Comput. Mol. Sci..

[177] Noid W., Szukalo R. J., Kidder K. M., Lesniewski M. C. (2024). Rigorous
progress in coarse-graining. Annu. Rev. Phys.
Chem..

[178] Guenza M. G. (2025). Everything you want to know about
coarse-graining and
never dared to ask: Macromolecules as a key example. Wiley Interdiscip. Rev.: Comput. Mol. Sci..

[179] Harmandaris V. A., Kremer K. (2009). Predicting polymer dynamics at multiple
length and time scales. Soft Matter.

[180] Ye H., Xian W., Li Y. (2021). Machine learning
of coarse-grained
models for organic molecules and polymers: Progress, opportunities,
and challenges. ACS Omega.

[181] Noid W. G. (2023). Perspective: Advances, challenges,
and insight for
predictive coarse-grained models. J. Phys. Chem.
B.

[182] Vettorel T., Besold G., Kremer K. (2010). Fluctuating soft-sphere
approach to coarse-graining of polymer models. Soft Matter.

[183] Español P., Serrano M., Pagonabarraga I., Zúñiga I. (2016). Energy-conserving
coarse-graining of complex molecules. Soft Matter.

[184] Husic, B. E. ; Charron, N. E. ; Lemm, D. ; Wang, J. ; Pérez, A. ; Majewski, M. ; Krämer, A. ; Chen, Y. ; Olsson, S. ; De Fabritiis, G. ; Noe, F. ; Clementi, C. Coarse graining molecular dynamics with graph neural networks J. Chem. Phys. 2020; Vol. 153, 10.1063/5.0026133. PMC7671749 33218238

[185] Wang X., Wu Y., Zhang A., He X., Chua T.-S. (2021). Towards multi-grained explainability for graph neural
networks. Adv. Neural Inf. Process. Syst..

[186] Peter C., Kremer K. (2009). Multiscale simulation
of soft matter
systems-from the atomistic to the coarse-grained level and back. Soft Matter.

[187] Zhao, Y. ; Chen, G. ; Liu, J. Polymer data challenges in the ai era: Bridging gaps for next-generation energy materials, 2025, arXiv:2505.13494. arXiv.org e-Print archive https://arxiv.org/abs/2505.13494.

[188] Orio M., Pantazis D. A., Neese F. (2009). Density functional
theory. Photosynth. Res..

[189] Joshi, S. P. ; Bucsek, A. ; Pagan, D. C. ; Daly, S. ; Ravindran, S. ; Marian, J. ; Bessa, M. A. ; Kalidindi, S. R. ; Admal, N. C. ; Reina, C. ; Ghosh, S. ; Warren, J. A. ; Vinals, J. ; Tadmor, E. B. “Integrated experiment and simulation co-design: A key infrastructure for predictive mesoscale materials modeling, 2025, arXiv:2503.09793. arXiv.org e-Print archive https://arxiv.org/abs/2503.09793.

[190] Zaporozhets I., Clementi C. (2023). Multibody terms in protein coarse-grained
models: A top-down perspective. J. Phys. Chem.
B.

[191] Jin J., Pak A. J., Durumeric A. E., Loose T. D., Voth G. A. (2022). Bottom-up
coarse-graining: Principles and perspectives. J. Chem. Theory Comput..

[192] Praprotnik M., Delle Site L., Kremer K. (2008). Multiscale simulation
of soft matter: From scale bridging to adaptive resolution. Annu. Rev. Phys. Chem..

[193] Milano, G. ; Kawakatsu, T. Hybrid particle-field molecular dynamics simulations for dense polymer systems J. Chem. Phys. 2009; Vol. 130, 10.1063/1.3142103. 19508055

[194] Wang J., Han Y., Xu Z., Yang X., Ramakrishna S., Liu Y. (2021). Dissipative particle dynamics simulation:
A review on investigating mesoscale properties of polymer systems. Macromol. Mater. Eng..

[195] Santo K. P., Neimark A. V. (2021). Dissipative particle dynamics simulations
in colloid and interface science: A review. Adv. Colloid Interface Sci..

[196] Hoogerbrugge P. J., Koelman J. (1992). Simulating microscopic
hydrodynamic
phenomena with dissipative particle dynamics. Europhys. Lett..

[197] Español P., Warren P. (1995). Statistical mechanics
of dissipative
particle dynamics. Europhys. Lett..

[198] Marrink S. J., Monticelli L., Melo M. N., Alessandri R., Tieleman D. P., Souza P. C. (2023). Two decades
of martini: Better beads,
broader scope. Wiley Interdiscip. Rev.: Comput.
Mol. Sci..

[199] Alessandri R., Grünewald F., Marrink S. J. (2021). The martini model
in materials science. Adv. Mater..

[200] Souza P. C. T., Alessandri R., Barnoud J., Thallmair S., Faustino I., Grünewald F., Patmanidis I., Abdizadeh H., Bruininks B. M., Wassenaar T. A., Kroon P., Melcr K., Nieto V., Corradi V., Khan H. M., Domanski J., Javanainen M., Martinez-Seara H., Reuter N., Best R. B., Vattulainen I., Monticelli L., Periole X., Peter T. D., Vries dH., Marrink S. J. (2021). Martini 3: a general purpose force field for coarse-grained
molecular dynamics. Nat. Methods.

[201] Fredrickson, G. H. The Equilibrium Theory of Inhomogeneous Polymers. Oxford University Press, 2006.

[202] Schmid F. (1998). Self-consistent-field theories for complex fluids. J. Phys.: Condens. Matter.

[203] Maerzke K. A., Siepmann J. I. (2011). Transferable potentials
for phase
equilibria- coarse-grain description for linear alkanes. J. Phys. Chem. B.

[204] Liwo A., Ołdziej S., Pincus M. R., Wawak R. J., Rackovsky S., Scheraga H. A. (1997). A united-residue force field for
off-lattice protein-structure simulations. i. functional forms and
parameters of long-range side-chain interaction potentials from protein
crystal data. J. Comput. Chem..

[205] Maisuradze G. G., Senet P., Czaplewski C., Liwo A., Scheraga H. A. (2010). Investigation of protein folding
by coarse-grained molecular dynamics with the unres force field. J. Phys. Chem. A.

[206] Reith D., Pütz M., Müller-Plathe F. (2003). Deriving effective
mesoscale potentials from atomistic simulations. J. Comput. Chem..

[207] Agrawal V., Arya G., Oswald J. (2014). Simultaneous
iterative
boltzmann inversion for coarse-graining of polyurea. Macromolecules.

[208] Tschöp W., Kremer K., Batoulis J., Bürger T., Hahn O. (1998). Simulation of polymer melts. i. coarse-graining
procedure for polycarbonates. Acta Polym..

[209] McCoy J. D., Curro J. G. (1998). Mapping of explicit
atom onto united
atom potentials. Macromolecules.

[210] Baschnagel J., Binder K., Doruker P., Gusev A. A., Hahn O., Kremer K., Mattice W. L., Müller-Plathe F., Murat M., Paul W., Santos S., Suter U. W., Tries V. (2000). Bridging the
gap between atomistic and coarse-grained models of polymers:
Status and perspectives. Viscoelasticity, Atomistic
Models, Statistical Chemistry.

[211] Akkermans R. L. C., Briels W. J. (2001). A structure-based coarse-grained
model for polymer melts. J. Chem. Phys..

[212] Uneyama T., Masubuchi Y. (2021). Plateau moduli
of several single-chain
slip-link and slip-spring models. Macromolecules.

[213] Behbahani A. F., Schneider L., Rissanou A., Chazirakis A., Bacova P., Jana P. K., Li W., Doxastakis M., Polinska P., Burkhart C., Muller M., Harmandaris V. A. (2021). Dynamics
and rheology of polymer melts via hierarchical atomistic, coarse-grained,
and slip-spring simulations. Macromolecules.

[214] Wu Z., Müller-Plathe F. (2022). Slip-spring
hybrid particle-field
molecular dynamics for coarse-graining branched polymer melts: Polystyrene
melts as an example. J. Chem. Theory Comput..

[215] Underhill P. T., Doyle P. S. (2004). On the coarse-graining of polymers
into bead-spring chains. J. Non-Newtonian Fluid
Mech..

[216] Kremer K., Grest G. S. (1990). Dynamics of entangled linear polymer
melts: A molecular-dynamics simulation. J. Chem.
Phys..

[217] Everaers R., Karimi-Varzaneh H. A., Fleck F., Hojdis N., Svaneborg C. (2020). Kremer-grest
models for commodity polymer melts: Linking
theory, experiment, and simulation at the kuhn scale. Macromolecules.

[218] Nitta, H. ; Ozawa, T. ; Yasuoka, K. Construction of full-atomistic polymer amorphous structures using reverse-mapping from kremer–grest models J. Chem. Phys., 2023 159 no. 19. 10.1063/5.0159722 37982485

[219] Miwatani R., Takahashi K. Z., Arai N. (2020). Performance of coarse
graining in estimating polymer properties: Comparison with the atomistic
model. Polymers.

[220] Praprotnik M., Delle Site L., Kremer K. (2006). Adaptive resolution
scheme for efficient hybrid atomistic-mesoscale molecular dynamics
simulations of dense liquids. Phys. Rev. E.

[221] Darré L., Machado M. R., Brandner A. F., González H. C., Ferreira S., Pantano S. (2015). Sirah: a structurally
unbiased coarse-grained
force field for proteins with aqueous solvation and long-range electrostatics. J. Chem. Theory Comput..

[222] Das R., Baker D. (2008). Macromolecular modeling
with rosetta. Annu. Rev. Biochem..

[223] Leman J. K., Weitzner B. D., Lewis S. M., Adolf-Bryfogle J., Alam N., Alford R. F., Aprahamian M., Baker D., Barlow K. A., Barth P., Basanta B., Bender B. J., Blacklock K., Bonet J., Boyken S. E., Bradley P., Bystroff C., Conway P., Cooper S., Correia B. E., Coventry B., Das R., De Jong R. M., DiMaio F., Dsilva L., Dunbrack R., Ford A. S., Frenz B., Fu D. Y., Geniesse C., Goldschmidt L., Gowthaman R., Gray J. J., Gront D., Guffy S., Horowitz S., Huang P.-S., Huber T., Jacobs T. M., Jeliazkov J. R., Johnson D. K., Kappel K., Karanicolas J., Khakzad H., Khar K. R., Khare S. D., Khatib F., Khramushin A., King I. C., Kleffner R., Koepnick B., Kortemme T., Kuenze G., Kuhlman B., Kuroda D., Labonte J. W., Lai J. K., Lapidoth G., Leaver-Fay A., Lindert S., Linsky T., London N., Lubin J. H., Lyskov S., Maguire J., Malmström L., Marcos E., Marcu O., Marze N. A., Meiler J., Moretti R., Mulligan V. K., Nerli S., Norn C., Ó’Conchúir S., Ollikainen N., Ovchinnikov S., Pacella M. S., Pan X., Park H., Pavlovicz R. E., Pethe M., Pierce B. G., Pilla K. B., Raveh B., Renfrew P. D., Roy Burman S. S., Rubenstein A., Sauer M. F., Scheck A., Schief W., Schueler-Furman O., Sedan Y., Sevy A. M., Sgourakis N. G., Shi L., Siegel J. B., Silva D.-A., Smith S., Song Y., Stein A., Szegedy M., Teets F. D., Thyme S. B., Wang R. Y.-R., Watkins A., Zimmerman L., Bonneau R. (2020). Macromolecular modeling and design in rosetta: recent
methods and frameworks. Nat. Methods.

[224] Baldassarre, F. ; Azizpour, H. Explainability techniques for graph convolutional networks, 2019, arXiv:1905.13686 arXiv.org e-Print archive https://arxiv.org/abs/1905.13686.

[225] Bai Y., Wilbraham L., Slater B. J., Zwijnenburg M. A., Sprick R. S., Cooper A. I. (2019). Accelerated discovery of organic
polymer photocatalysts for hydrogen evolution from water through the
integration of experiment and theory. J. Am.
Chem. Soc..

[226] Vosko S. H., Wilk L., Nusair M. (1980). Accurate spin-dependent
electron liquid correlation energies for local spin density calculations:
a critical analysis. Can. J. Phys..

[227] Becke A.
D. (1993). Density-functional thermochemistry.
iii. the role of
exact exchange. J. Chem. Phys..

[228] Lee C., Yang W., Parr R. G. (1988). Development
of the colle-salvetti
correlation-energy formula into a functional of the electron density. Phys. Rev. B.

[229] Grimme S., Bannwarth C., Shushkov P. (2017). A robust and accurate
tight-binding quantum chemical method for structures, vibrational
frequencies, and noncovalent interactions of large molecular systems
parametrized for all spd-block elements (z= 1–86). J. Chem. Theory Comput..

[230] Stephens P. J., Devlin F. J., Chabalowski C. F., Frisch M. J. (1994). Ab initio calculation of vibrational absorption and
circular dichroism spectra using density functional force fields. J. Phys. Chem. A.

[231] Yang K., Swanson K., Jin W., Coley C., Eiden P., Gao H., Guzman-Perez A., Hopper T., Kelley B., Mathea M. (2019). Analyzing
learned molecular representations for property prediction. J. Chem. Inf. Model..

[232] Rogers D., Hahn M. (2010). Extended-connectivity
fingerprints. J. Chem. Inf. Model..

[233] Landrum, G. Rdkit: open-source cheminformatics from machine learning to chemical registration. In Abstracts of Papers of the American Chemical Society; Vol. 258, AMER CHEMICAL SOC 1155 16TH ST, NW, WASHINGTON, DC 20036 USA, 2019.

[234] Mannodi-Kanakkithodi A., Chandrasekaran A., Kim C., Huan T. D., Pilania G., Botu V., Ramprasad R. (2018). Scoping the
polymer genome: A roadmap for rational polymer dielectrics design
and beyond. Mater. Today.

[235] Kim C., Chandrasekaran A., Huan T. D., Das D., Ramprasad R. (2018). Polymer genome:
A data-powered polymer informatics
platform for property predictions. J. Phys.
Chem. C.

[236] Ward L., Dunn A., Faghaninia A., Zimmermann N. E., Bajaj S., Wang Q., Montoya J., Chen J., Bystrom K., Dylla M., Chard K., Asta M., Persson K. A., Snyder G. J., Foster I., Jain A. (2018). Matminer:
An open source toolkit for materials data mining. Comput. Mater. Sci..

[237] Lightstone, J. P. ; Chen, L. ; Kim, C. ; Batra, R. ; Ramprasad, R. Refractive index prediction models for polymers using machine learning J. Appl. Phys. 2020; Vol. 127, 10.1063/5.0008026.

[238] Chen L., Kim C., Batra R., Lightstone J. P., Wu C., Li Z., Deshmukh A. A., Wang Y., Tran H. D., Vashishta P., Sotzing G. A., Cao Y., Ramprasad R. (2020). Frequency-dependent
dielectric constant prediction of polymers using machine learning. npj Computat. Mater..

[239] Doan Tran H., Kim C., Chen L., Chandrasekaran A., Batra R., Venkatram S., Kamal D., Lightstone J. P., Gurnani R., Shetty P. (2020). Machine-learning predictions
of polymer properties with polymer genome. J.
Appl. Phys..

[240] Godwin, J. ; Schaarschmidt, M. ; Gaunt, A. ; Sanchez-Gonzalez, A. ; Rubanova, Y. ; Veličković, P. ; Kirkpatrick, J. ; Battaglia, P. Simple gnn regularisation for 3d molecular property prediction & beyond, 2021, arXiv:2106.07971. arXiv.org e-Print archive https://arxiv.org/abs/2106.07971.

[241] Chen D., Lin Y., Li W., Li P., Zhou J., Sun X. (2020). Measuring and relieving the over-smoothing
problem for graph neural networks from the topological view. Proc. AAAI Conf. Artificial Intelligence.

[242] Frisch, M. Gaussian 09, Revision D.01, Gaussian, 2009.

[243] Kuenneth C., Schertzer W., Ramprasad R. (2021). Copolymer
informatics with multitask deep neural networks. Macromolecules.

[244] You, J. ; Ying, Z. ; Leskovec, J. Design space for graph neural networks. In Advances in Neural Information Processing Systems (NeurIPS) 2020. PMC7138248 32265580

[245] Rupp M., Tkatchenko A., Müller K.-R., Von Lilienfeld O. A. (2012). Fast and accurate modeling of molecular atomization
energies with machine learning. Phys. Rev. Lett..

[246] Bartók A. P., Kondor R., Csányi G. (2013). On representing
chemical environments. Phys. Rev. B.

[247] Townsend J., Micucci C. P., Hymel J. H., Maroulas V., Vogiatzis K. D. (2020). Representation of molecular structures
with persistent
homology for machine learning applications in chemistry. Nat. Commun..

[248] Huo H., Rupp M. (2022). Unified representation of molecules and crystals for
machine learning. Machine Learning: Sci. Technol..

[249] Sanchez Medina E. I., Linke S., Stoll M., Sundmacher K. (2023). Gibbs-helmholtz
graph neural network: capturing the temperature dependency of activity
coefficients at infinite dilution. Digital Discovery.

[250] Zhong C., Sato Y., Masuoka H., Chen X. (1996). Improvement
of predictive accuracy of the unifac model for vapor-liquid equilibria
of polymer solutions. Fluid Phase Equilib..

[251] Kontogeorgis G. M., Fredenslund A., Tassios D. P. (1993). Simple activity
coefficient model for the prediction of solvent activities in polymer
solutions. Ind. Eng. Chem. Res..

[252] Pappa G. D., Voutsas E. C., Tassios D. P. (1999). Prediction
of activity
coefficients in polymer and copolymer solutions using simple activity
coefficient models. Ind. Eng. Chem. Res..

[253] Cremer D. (2011). Møller-plesset perturbation
theory: from small
molecule methods to methods for thousands of atoms. Wiley Interdiscip. Rev.: Comput. Mol. Sci..

[254] Kearnes S., McCloskey K., Berndl M., Pande V., Riley P. (2016). Molecular graph convolutions:
moving beyond fingerprints. J. Comput.-Aided
Mol. Des..

[255] Wang, M. Y. Deep graph library: Towards efficient and scalable deep learning on graphs. In ICLR Workshop on Representation Learning on Graphs and Manifolds, 2019.

[256] Bojar D., Powers R. K., Camacho D. M., Collins J. J. (2021). Deep-learning
resources for studying glycan-mediated host-microbe interactions. Cell Host Microbe.

[257] Montavon, G. ; Binder, A. ; Lapuschkin, S. ; Samek, W. ; Müller, K.-R. Layer-wise relevance propagation: an overview. In Lecture Notes in Computer Science 2019; pp 193–209.

[258] Binder, A. ; Bach, S. ; Montavon, G. ; Müller, K.-R. ; Samek, W. Layer-wise relevance propagation for deep neural network architectures. In Information Science and Applications (ICISA) 2016; Springer, 2016; pp 913–922.

[259] Adebayo, J. ; Gilmer, J. ; Muelly, M. ; Goodfellow, I. ; Hardt, M. ; Kim, B. Sanity checks for saliency maps Advances in Neural Information Processing Systems, 2018 31.

[260] Qi Z., Khorram S., Li F. (2020). Visualizing
deep networks by optimizing
with integrated gradients. AAAI.

